# Supplementary material for: Harnessing microbial-derived metabolites in the urinary tract to prevent infection induced catheter encrustation
Source: Nat Commun. 2025 Nov 3;16:9678. doi: 10.1038/s41467-025-64661-y (PMC12583827; doi:10.1038/s41467-025-64661-y)
Supplement: Supplementary file 1 — Supplementary information [file 41467_2025_64661_MOESM1_ESM.pdf]

Title: Harnessing microbial-derived metabolites in the urinary tract to prevent infection induced catheter encrustation

One sentence summary: Uropathogens secrete metabolites that modulate bacterial urease activity and can be used to prevent infection-induced catheter encrustation.

Authors: L. Beryl Guterman[1] (e: laurengu@buffalo.edu), Madalyn Motsay[1] (e: msmotsay@gmail.com), Benjamin C. Hunt[1] (e: bchunt@buffalo.edu), Aimee L. Brauer [1] (e: albrauer@buffalo.edu), Brian S. Learman[1] (e: bslearma@buffalo.edu), Mindula K. Wijayahena[2] (e: mindulaw@buffalo.edu), Alexander C. Hoepker[2,3] (e: ahoepker@buffalo.edu), Diana S. Aga[2,3] (e: dianaaga@buffalo.edu), Brittany Francis[1] (e: brittanykf101@gmail.com), Beatriz M. Fontoura[4] (e: fontoub@wfu.edu), George L. Donati[4*] (e: George.Donati@health.ny.gov), Peter J. Bush[5] (e: pjbush@buffalo.edu), Namrata Deka[1] (e: ndeka@buffalo.edu) and Chelsie E. Armbruster[1†] (e: chelsiea@buffalo.edu)

Affiliations:

[1]Department of Microbiology and Immunology, Jacobs School of Medicine and Biomedical Sciences, State University of New York at Buffalo, Buffalo, NY 14203, United States

[2]Department of Chemistry, University at Buffalo, The State University of New York, Buffalo, NY 14260, United States

[3]Research and Education in Energy, Environment and Water (RENEW), University at Buffalo, The State University of New York, Buffalo, NY 14260, United States

[4]Department of Chemistry, Wake Forest University, Winston Salem, NC 27109, United States

[5]Laboratory for Forensic Odontology Research, School of Dental Medicine, SUNY at Buffalo, B1 Squire Hall, S. Campus, Buffalo, NY 14214, United States

*Current address: Laboratory of Inorganic and Nuclear Chemistry, Wadsworth Center, New York State Department of Health, Albany, NY 12237, United States.

†Corresponding author: Chelsie E. Armbruster, chelsiea@buffalo.edu

List of Supplementary Materials

Supplementary Methods and Materials

Supplementary Figure 1. *P. mirabilis* urease activity during incubation in each of the monomicrobial cell-free saline supernatants.

Supplementary Figure 2. *P. mirabilis* urease activity during incubation in candidate dampening metabolites.

Supplementary Figure 3. Differences between lots of imidazole lactate in structure, physical appearance and impact on *P. mirabilis* urease activity.

Supplementary Figure 4. 1H-NMR spectra of D-imidazole lactate and L imidazole lactate.

Supplementary Figure 5. Growth and viability of *P. mirabilis* incubated in dampening metabolites.

Supplementary Figure 6. All metabolites except phenylpyruvate decrease urease activity in *P. mirabilis* cell free extracts.

Supplementary Figure 7. Kinetics of inhibition of urease activity in cell free extracts of *P. mirabilis* by Histamine HCl (His).

Supplementary Figure 8. Kinetics of inhibition of urease activity in cell free extracts of *P. mirabilis* by leucylglycine (LeuGly).

Supplementary Figure 9. Kinetics of inhibition of urease activity in cell free extracts of *P. mirabilis* by D-imidazole lactate (DIL).

Supplementary Figure 10. Kinetics of inhibition of urease activity in cell free extracts of *P. mirabilis* by L-imidazole lactate (LIL).

Supplementary Figure 11. Kinetics of inhibition of urease activity in cell free extracts of *P. mirabilis* by imidazole (Imid).

Supplementary Figure 12. Kinetics of inhibition of urease activity in cell free extracts of *P. mirabilis* by 4-imidazole acetate (4IA).

Supplementary Figure 13. Microbial metabolites dampen activity of purified Jack Bean Urease (JBU).

Supplementary Figure 14. JBU activity during incubation in candidate dampening metabolites.

Supplementary Figure 15. Activity of Jack bean urease (JBU) incubated in high concentrations (>20mM) of Histamine HCl and Phenylpyruvate.

Supplementary Figure 16. RNA transcript stability and expression profiles of *P. mirabilis* urease operon and housekeeping gene.

Supplementary Figure 17. Urease activity of *P. mirabilis* 104V0, 106V15 and HU1069 incubated in urease-dampening metabolites.

Supplementary Figure 18. Multiple sequence alignment of urease protein subunits across three *Proteus mirabilis* isolates.

Supplementary Figure 19. Growth of clinical isolates of *P. mirabilis* incubated in dampening metabolites.

Supplementary Figure 20. Growth of *M. morganii, P. stuartii* and UTI MRSA incubated in dampening metabolites.

Supplementary Figure 21. Microbial metabolites dampen *P. mirabilis* urease activity in human urine.

Supplementary Figure 22. Urease-dampening metabolites are non-cytotoxic.

Supplementary Figure 23. Synergistic effects of Histamine or 4-imidazole acetate, in combination with AHA and alone, on *P. mirabilis* urease activity in artificial urine media (AUM) supplemented with 500mM urea.

Supplementary Figure 24. Dose response of pH and bacterial CFUs to AHA, histamine, and 4-imidazole acetate in the *in vitro* CAUTI model.

Supplementary Figure 25. Measuring bacterial crystalline biofilm biomass and bacterial viability on 10 mm catheter segments 24 hours post-inoculation.

Supplementary Figure 26. Elemental composition of crystalline biofilm measured by energy dispersive spectroscopy (EDS).

Supplementary Methods

Cell free supernatant sample preparation for untargeted metabolomics. To remove protein, dissociate small molecules bound to protein or trapped in the precipitated protein matrix, and to recover chemically diverse metabolites, proteins were precipitated with methanol under vigorous shaking for 2 min (Glen Mills GenoGrinder 2000) followed by centrifugation. The resulting extract was divided into four fractions: two for analysis by separate reverse phase (RP)/UPLC-MS/MS methods with positive ion mode electrospray ionization (ESI), one for analysis by RP/UPLC-MS/MS with negative ion mode ESI, and one for analysis by HILIC/UPLC-MS/MS with negative ion mode ESI. All methods utilized a Waters ACQUITY ultra-performance liquid chromatography (UPLC) and a Thermo Scientific Q-Exactive high resolution/accurate mass spectrometer interfaced with a heated electrospray ionization (HESI-II) source and Orbitrap mass analyzer operated at 35,000 mass resolution.

Samples were placed briefly on a TurboVap® (Zymark) to remove the organic solvent, then dried and reconstituted in solvents compatible to each of the four methods. Each reconstitution solvent contained a series of standards at fixed concentrations to ensure injection and chromatographic consistency. One aliquot was analyzed using acidic positive ion conditions, chromatographically optimized for more hydrophilic compounds. In this method, the extract was gradient-eluted from a C18 column (Waters UPLC BEH C18-2.1x100 mm, 1.7 µm) using water and methanol, containing 0.05% perfluoropentanoic acid (PFPA) and 0.1% formic acid (FA). Another aliquot was also analyzed using acidic positive ion conditions, but chromatographically optimized for more hydrophobic compounds. In this method, the extract was gradient-eluted from the C18 column using methanol, acetonitrile, water, 0.05% PFPA and 0.01% FA and was operated at an overall higher organic content. Another aliquot was analyzed using basic negative ion

optimized conditions using a separate dedicated C18 column. The basic extracts were gradient-eluted from the column using methanol and water with 6.5mM Ammonium Bicarbonate at pH 8. The fourth aliquot was analyzed via negative ionization following elution from a HILIC column (Waters UPLC BEH Amide 2.1x150 mm, 1.7 µm) using a gradient consisting of water and acetonitrile with 10mM Ammonium Formate, pH 10.8. The MS analysis alternated between MS and data-dependent MS^n scans using dynamic exclusion. The scan range varied slightly between methods but covered 70-1000 m/z.

Raw data were extracted, peak-identified, and QC processed using Metabolon's hardware and software. Compounds were identified by comparison to library entries of more than 3300 commercially available purified standard compounds as well as recurrent unknown entities. The Metabolon library includes retention time/index (RI), mass to charge ratio (*m/z)*, and chromatographic data (including MS/MS spectral data) for all entries.

Peaks were quantified using area-under-the-curve. In certain instances, biochemical data were normalized to an additional factor (e.g., total protein as determined by Bradford assay, osmolality) to account for differences in metabolite levels due to differences in the amount of material present in each sample. Using these mean scaled intensities, fold change was calculated as the ratio of the mean scaled intensity for a metabolite between two experimental groups (Sup. Table 7). p-values for each comparison were derived from the Natural Log-Transformed fold change using one-way ANOVA with Tukey's test for multiple comparison (q=false discovery rate).

LC-HRMS analysis. The LC-HRMS analysis was performed in a Thermo Scientific Q-Exactive™ Focus Orbitrap™ LC-MS (Waltham, MA) with Dionex UltiMate™ 3000 ultra-HPLC system, under a full-scan data dependent MS2 (ddMS2) data acquisition method; a resolution was set at 17,500 and a scan range set at 50-1000 m/z. Chromatographic separation was achieved using a 2.1 x 100 mm Restek Raptor Polar X column with a particle size of 2.7 µm (Bellefonte, PA), with an isocratic mobile phase using 15 % mobile phase A (0.5% formic acid in water, pH 3) and 85 % mobile phase B (acetonitrile) for 40 min. An injection volume of 10 µL was used. The standards were analyzed in positive mode electrospray ionization (ESI). In all analyses, the protonated precursor ion $[M+H]^+$ for imidazole lactate was observed at m/z 157.0609 and no other significant peaks were present, suggesting that both lots are imidazole lactate (Supplementary. Fig. 3C, D).

Polarimetry. The specific optical rotation of 1.0 mg/mL and 0.5 mg/mL for L-imidazole lactate and D-imidazole lactate standards were measured with an Autopol 1 Automatic Polarimeter (Rudolph Research Analytical) using a 100 mm quartz cell at 22 °C and 589 nm. Optical rotation was remeasured in a DMSO/water/methanol mixture (45:25:30) and gave -0.10 and +0.08 for lots 1 and 2, respectively, suggesting that the two lots are optical isomers (enantiomers): a clockwise (+) rotation indicates the "D" version of the compound, while a counterclockwise (-) rotation indicates the "L" version of the compound (*60, 61*).

^{1}H-NMR spectroscopy. All 1H NMR spectra were recorded on a Bruker Avance Neo 500 MHz NMR spectrometer. The variable temperature 1H-NMR (VT 1H-NMR) experiments were recorded at 25 ºC, 40 ºC and 50 ºC at 500 MHz. One set of NMR samples was prepared in 100 % of the solvent dimethyl sulfoxide-d6 (DMSO-d6) and the second set was prepared in deuterium dioxide (D2O) by obtaining an aliquot of DMSO-d6 dissolved standards to have 30 % of

DMSO-d6 and 70 % of D2O. The 1H resonances were referenced to the CHD2 resonance (pentuplet) of DMSO- d6 at 2.50 ppm and the D2O resonance at 4.79 ppm. The 1H-NMR spectra at 25ºC also confirmed that both lots are imidazole lactate (Supplementary. Fig. 4A). The 1H NMR spectra confirmed the presence of two distinct forms of imidazole lactate arising from the combination of the hindered rotation of the imidazole ring, as evidenced by variable-temperature 1H-NMR experiments (Supplementary. Table 4), that is likely assisted by intramolecular H-bonding and the chiral optically active proton (H4) on the alcoholic carbon (Supplementary. Fig. 4 A, B).

Growth curves. Bacterial strains were cultured for ~18 hours in LB broth or BHI, then diluted 1:100 into fresh media with or without dampening metabolites. Bacterial suspensions were distributed into wells of a 96-well plate, incubated at 37ºC with double-orbital shaking, and OD_{600} was measured every 15 minutes for 18 hours in a Synergy H1 plate reader (BioTek). All growth curves were conducted in triplicate with at least three biological replicates.

Tolerance assay. Bacteria were cultured for ~18 hours in LB or BHI broth and then sub cultured to mid-log growth phase (OD_{600} 0.5), washed once in potassium phosphate buffer, and diluted 1:100 in D-imidazole lactate dissolved in either potassium phosphate buffer or filter-sterilized human urine (Cone Bioproducts, Sequin, TX). Bacterial suspensions were incubated at 37°C for 15 minutes or 1 hour at 225 rpm. Samples underwent serial 10-fold dilutions and were spiral plated (Eddy Jet 2; Neutec Group Inc., Farmingdale, NY) onto low-salt LB agar for enumeration of CFU using a ProtoCOL 3 automated colony counter (Synbiosis).

Real-time quantitative PCR analysis. The data were analyzed according to the Relative Quantification (RQ) method by Pfaffl et al 2001 (*56*) in which *P. mirabilis* potassium phosphate buffer without urea was considered as the 'Control' and *P. mirabilis* in potassium phosphate buffer supplemented with 500mM urea with and without candidate dampening compounds were the 'Sample.' The full equation is below.

$$\text{Relative Quantification Ratio} = \frac{(\mathrm{E_{target}})^{\Delta \mathrm{CPtarget\ (control-sample)}}}{(\mathrm{E_{reference}})^{\Delta \mathrm{CPreference\ (control-sample)}}} \qquad \text{Equation 2}$$

In this equation, E refers to the primer efficiency of the target gene (Etarget, urease operon subunits) or the reference gene (Ereference, *rpoA*), and CP refers to the Cycle Point, or the cycle number at which the signal exceeds the threshold.

Urease operon sequencing and alignment. Freezer stocks were streak-plated on MacConkey agar to isolate single colonies and genomic DNA was extracted using the DNeasy Blood and Tissue Kit (Qiagen, Germantown, Maryland, USA). Samples were sequenced by SeqCoast Genomics for whole genome sequencing on the Illumina NextSeq2000 platform. Read trimming, and run analytics were performed using Trimmomatic v0.39.0 (*62*). The processed reads were assembled using spades v3.15.5 (*63*) wrapped in Unicycler v0.5.0 (*64, 65*). Read mapping was done using Bowtie2 and SAMtools (wrapped in Unicycler) (*66, 67*). The complete genome assemblies that passed quality filtering were annotated using prokka v1.14.5 (*68*). Urease genes, including the full operon from ureA to ureR, were identified and extracted from the prokka annotated genomes. The extracted protein sequences were subjected to sequence alignment using Clustal

Omega and visualization was carried out using ESPript 3.0 (*69*), enabling comparison across different clinical isolates. Urease genes, including the full operon from ureA to ureR, were identified and extracted from the prokka annotated genomes. The extracted protein sequences were subjected to sequence alignment using Clustal Omega and visualization was carried out using ESPript 3.0 [8], enabling comparison across different clinical isolates.

<u>UTI MRSA urease activity assay.</u> The UTI MRSA urease assay was adapted from a recently-published semi quantitative assay to measure urease activity in bacterial species with low overall activity under standard conditions (*50*). Dampening metabolites were directly dissolved in urease broth (1 g tryptone (RPI; CAS No. 91079-40-2), 1 g D(+)-glucose (RPI; CAS No. 50-99-7), 2 g potassium phosphate monobasic anhydrous (VWR; Lot: 20C1356567), 5 g sodium chloride (Fisher Chemical; Lot 234834), 0.012 g Phenol red sodium salt (Sigma; cat #P4758-50G), in 1 L of MilliQ water, pH 5.5, sterilized via autoclaving). UTI MRSA strains from overnight cultures were harvested by centrifugation for 5 minutes at 10,000 g, washed with 1 volume of 1X Phosphate Buffered Saline (PBS), and resuspended in urease broth. Bacterial cultures were transferred to a 96-well plate with or without urea (Fisher Chemical; Lot 219153). The OD was measured at 415 nm, 560 nm, and 600 nm every 20 mins for 24 hours, shaking at 37°C using a BioTek Synergy|H1 microtiter plate reader. Urease activity was calculated by dividing the OD values at 560 nm by the OD values at 415 nm and the urease activity was then normalized by dividing the OD of each dampening metabolite condition grown in urease broth containing urea by the same dampening metabolite condition grown in urease broth without urea.

<u>*In vitro* “bladder” model.</u> A previously described glass “bladder” model was used, in which a 500 mL water-jacketed glass vessel (Chemglass CG-1929-06) is maintained at 37°C via a

circulating water bath, a Foley catheter is inserted and inflated, and artificial urine medium (AUM) is supplied into the "bladder" at a constant flow rate (0.75-1.0mL/min) through a peristaltic pump (*28, 57, 70*). For consistency with the *in vitro* urease activity assays and the physiologic range of urea in healthy human urine, AUM was supplemented with 500mM urea (*59*). Urease-dampening metabolites of interest were directly dissolved in AUM 500mM urea, adjusted to a pH of 5.8, and filter sterilized via a 0.2μm filter. To prepare the inoculum, the *P. mirabilis* strains were first subcultured to mid log phase in LB (OD_{600} of 0.2-0.5). Inocula were spun down to pellet at 10,000 rcf for 10min at 24°C and resuspended in 10mL of AUM 500mM urea with and without and urease-dampening metabolites such that the final OD_{600} was 1.0. All 10mL of the resulting inoculum was introduced directly into the water-jacketed glass vessel of a "bladder" set-up supplied with media containing the same metabolite (or AUM only control) and allowed to establish for 1 hour before initiating flow. Samples were then collected from the catheter port at 0, 3, 6, 9, 12, and 24 hours to monitor pH (METTLER TOLEDO™ SevenCompact™ S220 pH Benchtop Meter) and bacterial CFUs. After 24 hours, the catheters were carefully removed, catheter eyelets were dissected by cutting sections 2 cm in length from the catheter tip to immediately proximal to the balloon and stored in -80C for scanning electron microscopy. The remaining length of each catheter was then cut into three 9 cm sections (upper, middle, and lower) starting below the catheter balloon. Each section was further cut into nine 10 mm segments. Triplicate segments from each section were assessed for total biomass (crystal violet staining), bacterial viability (CFUs), and ion composition as detailed below.

Catheter segment biofilm biomass analysis. Residual AUM flow through was gently removed from the catheter lumen by wicking on a KimWipe (Kimberly-Clark Corporation, Loudon, TN),

and the segments were air dried for 15 min. Catheter segments were stained with 1.0 ml 0.1% crystal violet for 10 min, washed by gentle dunking in 1X phosphate-buffered saline (PBS) to remove excess stain, and solubilized in a microcentrifuge tube containing 750μL of 1% SDS 95% ethanol and vortexed for 5 minutes. Crystal violet absorbance (OD_{570}) was measured in a BioTek Synergy H1 microplate reader and blanked using cell-free control wells for each biofilm assay.

Catheter segment biofilm viability. Catheter segments were washed by gentle dunking in 1X phosphate-buffered saline (PBS) to remove any remaining planktonic bacteria and residual AUM from the catheter lumen. Catheter segments were then placed in a microcentrifuge tube containing 1mL of 1X PBS and vortexed for 5 minutes to solubilize the biofilm. Samples underwent serial 10-fold dilutions and were spiral plated (Eddy Jet 2; Neutec Group Inc., Farmingdale, NY) onto low-salt LB agar for enumeration of CFU using a ProtoCOL 3 automated colony counter (Synbiosis).

Catheter segment elemental composition. Catheter segments were placed in a microcentrifuge tube containing 1mL of Mili-Q water and vortexed for 5 minutes to solubilize the crystalline biofilm. Catheter segments were discarded, and the suspended/solubilized crystalline biofilms were pooled such that the material from three catheter segments per condition were combined for ICP-EOS analysis. Organic material was digested using trace metal grade nitric acid and heating at 100°C for 30 minutes. The samples were then diluted with distilled-deionized water in a centrifuge polypropylene tube (50-fold for untreated *P. mirabilis* conditions and 5-fold diluted for treated *P. mirabilis* and urease mutant conditions). The final acid concentration in solution

was 1% v/v HNO_3. The acid-digested catheter deposit samples were then analyzed by inductively coupled plasma optical emission spectrometry (ICP-OES). An Agilent 5110 ICP-OES (Agilent Technologies, Santa Clara, CA, USA) equipped with a SPS4 automatic sampler, concentric nebulizer, and double-pass cyclonic spray chamber was used in all determinations. High purity argon (99.999%, ARC3 Gases, South Dunn, NC, USA) was employed for plasma generation and sample solution nebulization and transport. Additional details on the ICP-OES operating conditions are shown in Supplementary Table 8. All solutions were prepared with distilled-deionized water (Milli-Q®, Millipore, Bedford, MA, USA) in 1% v/v HNO_3 trace metal grade medium (Fisher, Pittsburgh, PA, USA). Single-element reference solutions of Ca, Mg and P at 1000 mg/L (HPS, High-Purity Standards, North Charleston, SC, USA) were appropriately diluted to prepare the standard solutions used in external standard calibration (EC). (*71*) The calibration solutions used for EC were at 0.2, 0.5, 1.0, 2.0, and 5.0 mg/L for all analytes.

Due to relatively high analyte concentrations, samples were adequately diluted with distilled-deionized water (5-fold or 50-fold) before analysis by ICP-OES. Instrumental limits of detection (LOD) were calculated according to IUPAC recommendations as three times the standard deviation (S_{blk}) for fifteen measurements of the analytical blank (1% v/v HNO_3) divided by the slope of the calibration curve (m), i.e., LOD = $3S_{blk}/m$. (*72*) LOD values for Ca, Mg and P were 0.7, 0.1 and 8 μg/L, respectively. Addition and recovery experiments were also carried out to validate the method. Three samples (Pm, 5mM AHA, and 1.25mM AHA 12.5mM Histamine), were diluted 5-fold and spiked with the analytes at two concentration levels in this experiment. Spike concentrations were chosen based on LOD values and concentrations levels expected in the samples. Analyte percent recoveries ranged from 93.3% to 108% (Supplementary. Table 9).

Microscopy. The 2 cm catheter eyelet segments were prepared for electron microscopy by fixing in 2.5% glutaraldehyde (Election Microscopy Sciences, Hatfield, PA, USA) in 0.1M Sorensen buffer (Election Microscopy Sciences, Hatfield, PA, USA) pH 8 for 4 hours at room temperature. Sublimation and dehydration was then performed in an ascending ethanol series (i.e. 70%, 90%, 100%) diluted 1:1 in hexamethyldisilizane, 98% (HMDS, Thermo Fisher Scientific, Lancashire UK). Finally, they were washed twice in 100% HMDS (15 minutes each step) and left to dry in air overnight. Samples were first imaged using a stereomicroscope (Nikon SMZU stereomicroscope). The samples were then sectioned longitudinally and coated with evaporated carbon (Denton DV502) and the luminal surfaces were visualized using a Hitachi SU70 scanning electron microscope at an accelerating voltage of 2kV. To measure composition of biofilm on fixed catheter segments energy dispersive spectroscopy was used at 20kV.

Cell culture. HEK293 cells (American Type Culture Collection) and T24 (ATCC HTB-4) were resuscitated from frozen stocks (liquid N_2) in Dulbecco's Eagle Medium (DMEM) and Roswell Park Memorial Institute (RPMI) media, respectively, supplemented with fetal bovine serum (10% v/v) and cultured in controlled atmosphere (5% CO_2, 37°C). Adherent cells were routinely passaged in tissue culture treated petri dishes every 2-3 days or when the cells reached 70–80% confluency. Cells were detached by pre-washing in 9 mL DPBS to chelate divalent cations, followed by trypsinization (0.25%, 1 min). Cells were resuspended in fresh medium (DMEM/RPMI), counted using a hemocytometer, and diluted in DMEM/RPMI to achieve 1×10^5 cells/mL. Cells were then seeded in triplicate into 24-well plate (500 µL per well) and incubated overnight to form confluent monolayers.

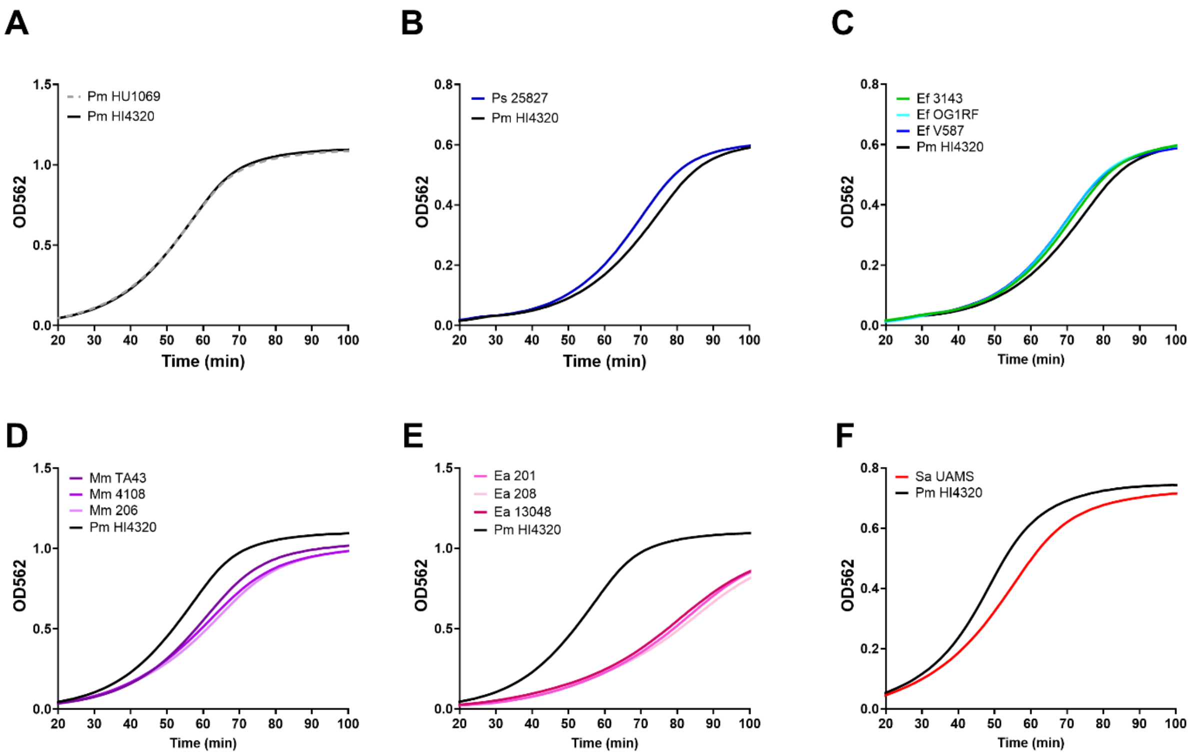

Supplementary Figure 1. *P. mirabilis* urease activity during incubation in each of the monomicrobial cell-free saline supernatants. Cell-free saline supernatants were generated from the following monomicrobial cultures of strains of interest: (A) *P. mirabilis* (Pm) HI4320 and HU1069 (B) *P. stuartii* (Ps) 25827 (C) *E. faecalis* (Ef) 3143, OG1RF and V587 (D) *M. morganii* (Mm) TA43, 4108 and 206 (E) *E. aerogenes* (Ea) 201, 208 and 13048 and (F) *S. aureus* UAMS. Cell-free saline supernatants were generated by 90-min incubation in filter-sterilized 0.9% saline followed by 3kD filtration. *P. mirabilis* was then incubated in each of the cell-free saline supernatants supplemented with excess urea, and urease activity was monitored at 60-s intervals over 100 min. Representative graphs show means from three technical replicates. Each condition was performed for three independent experiments with at least three replicates each.

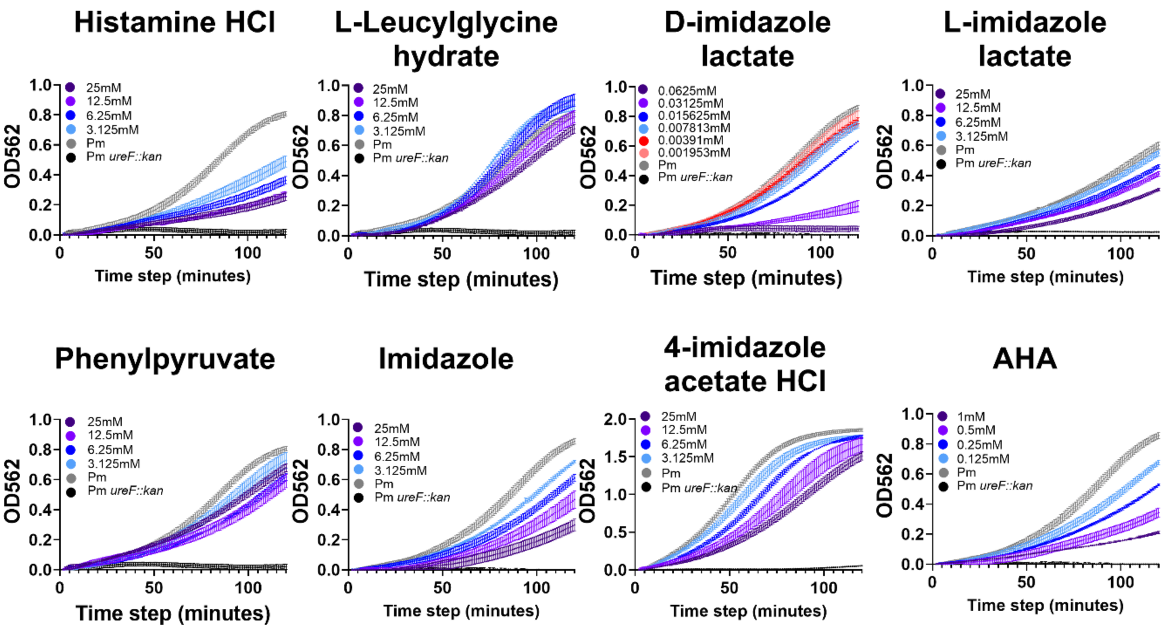

Supplementary Figure 2. *P. mirabilis* urease activity during incubation in candidate dampening metabolites. Representative dose-response curve of whole cell Pm urease activity when incubated in a candidate dampening metabolites or AHA, with a urease mutant (Pm *ureF::kan*) as a negative control. Representative graphs show means and standard deviations from three technical replicates.

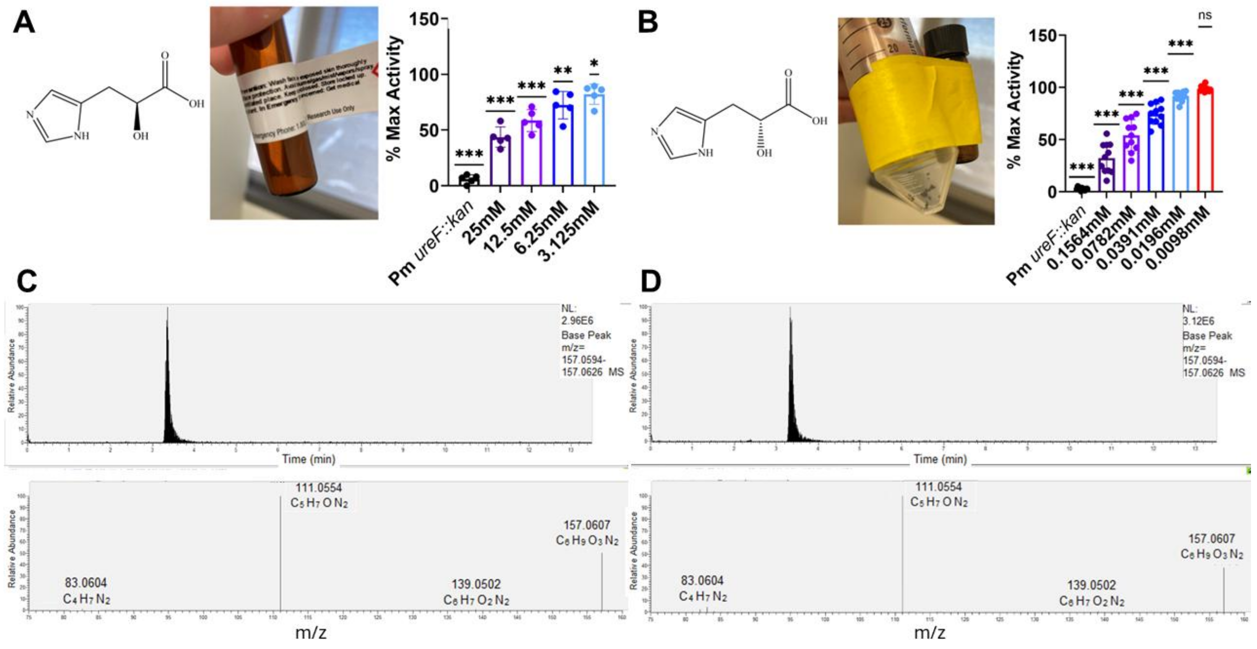

Supplementary Figure 3. Differences between lots of imidazole lactate in structure, physical appearance and impact on *P. mirabilis* urease activity. Differences in structure, physical appearance (left black sticky solid vs right loose white powder) and impact on *P. mirabilis* urease activity between (A) L-imidazole lactate and (B) D-imidazole lactate. Graphs show mean±SD for at least 3 independent experiments (L-imidazole lactate n=5 and D-imidazole lactate n=11) with 3 technical replicates each. Data were analyzed by two tailed one sample t-test to a hypothetical value of 100% activity. ***P<0.001, **P<0.002, *P<0.033. LC-HRMS chromatograms of L-imidazole lactate (C) and (D) D-imidazole lactate, showing the same retention times at 3.43 min, and protonated precursor ion $[M+H]^+$ at m/z 157.0609. The corresponding extracted MS/MS spectra also show the same major fragment ions at m/z 111.0554 and 83.0604, to be within less than a 5 ppm difference.

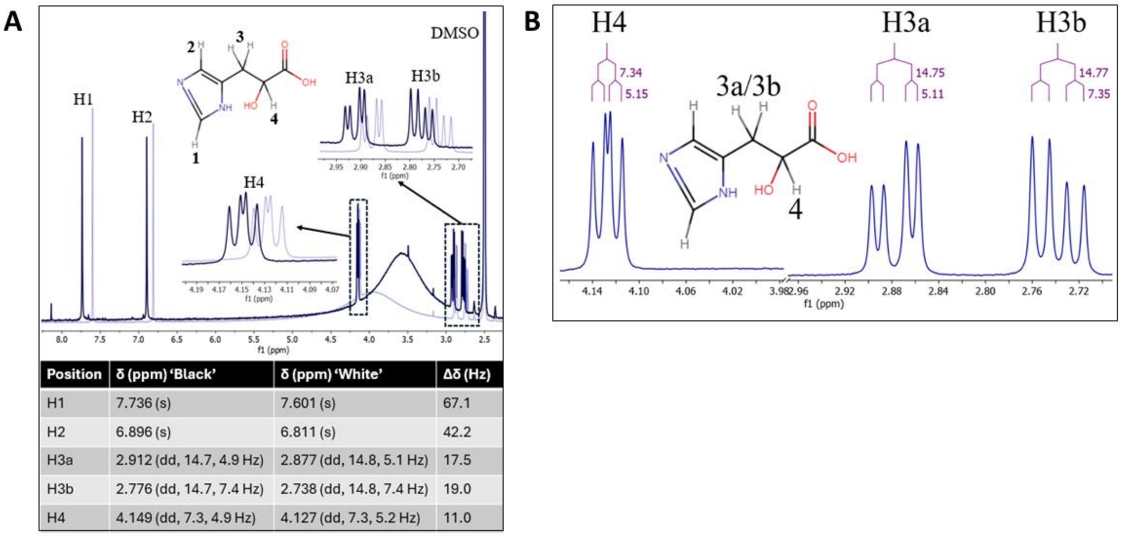

Position	δ (ppm) 'Black'	δ (ppm) 'White'	Δδ (Hz)
H1	7.736 (s)	7.601 (s)	67.1
H2	6.896 (s)	6.811 (s)	42.2
H3a	2.912 (dd, 14.7, 4.9 Hz)	2.877 (dd, 14.8, 5.1 Hz)	17.5
H3b	2.776 (dd, 14.7, 7.4 Hz)	2.738 (dd, 14.8, 7.4 Hz)	19.0
H4	4.149 (dd, 7.3, 4.9 Hz)	4.127 (dd, 7.3, 5.2 Hz)	11.0

Supplementary Figure 4. 1H-NMR spectra of D-imidazole lactate and L imidazole lactate.

(A) Superimposed 1H-NMR spectra at 25 ºC of Lot 1 (blue trace) and Lot2 (black trace) of imidazole lactate illustrating differences in chemical shift and J couplings. The broad peaks are likely water-exchangeable protons of the imidazole and alcohol groups. (B) ^{1}H-NMR (500 MHz spectrum of lot2 in DMSO-d6 showing the chemically distinct H3 protons each split by its geminal proton (14.8 Hz) and the H4 proton (5.1, 7.4 Hz). The splitting pattern of lot1 is similar with chemical shift and J- couplings shown in (A).

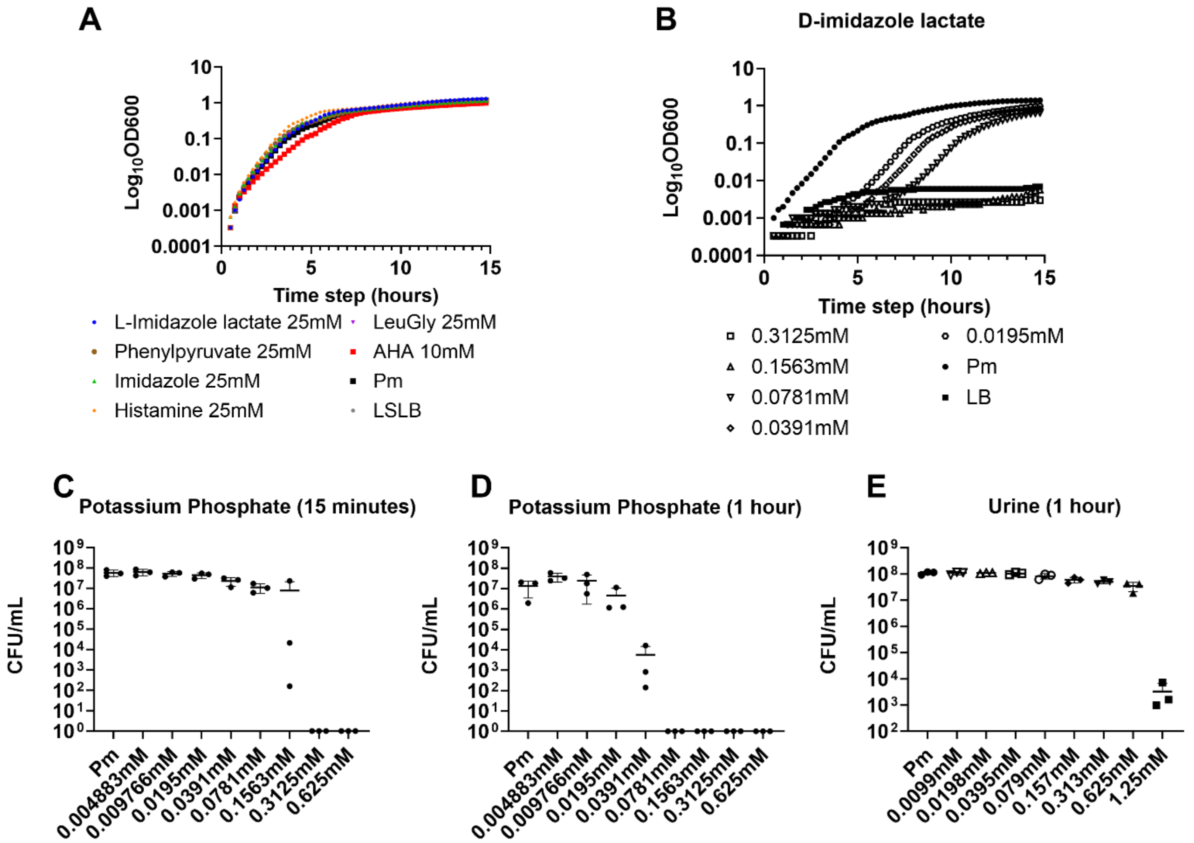

Supplementary Figure 5. Growth and viability of *P. mirabilis* incubated in dampening metabolites. (A-B) *P. mirabilis* was cultured for ~18 hours in LB broth, then diluted 1:100 into fresh LB broth with (A) the highest concentration of each dampening metabolites that was tested for urease-dampening activity or (B) serial dilutions of D-imidazole lactate. Bacterial suspensions were incubated at 37ºC with double-orbital shaking, and OD600 was measured every 15 minutes for 18 hours. Representative growth curves (A-B) show means and standard deviations from three technical replicates. (C-E) Viability of *P. mirabilis* incubated in serial dilutions of D-imidazole lactate. Overnight cultures were subcultured to mid log phase, washed in potassium phosphate buffer, and diluted 1:10 in either (C,D) potassium phosphate buffer or human urine (E) with and without D-imidazole lactate. Samples were incubated at 37°C with

aeration for either (C) fifteen minutes or (D,E) one hour then plated and enumerated for colony forming units (CFU). Data represent mean ± SD for three independent biologic replicates (n=3).

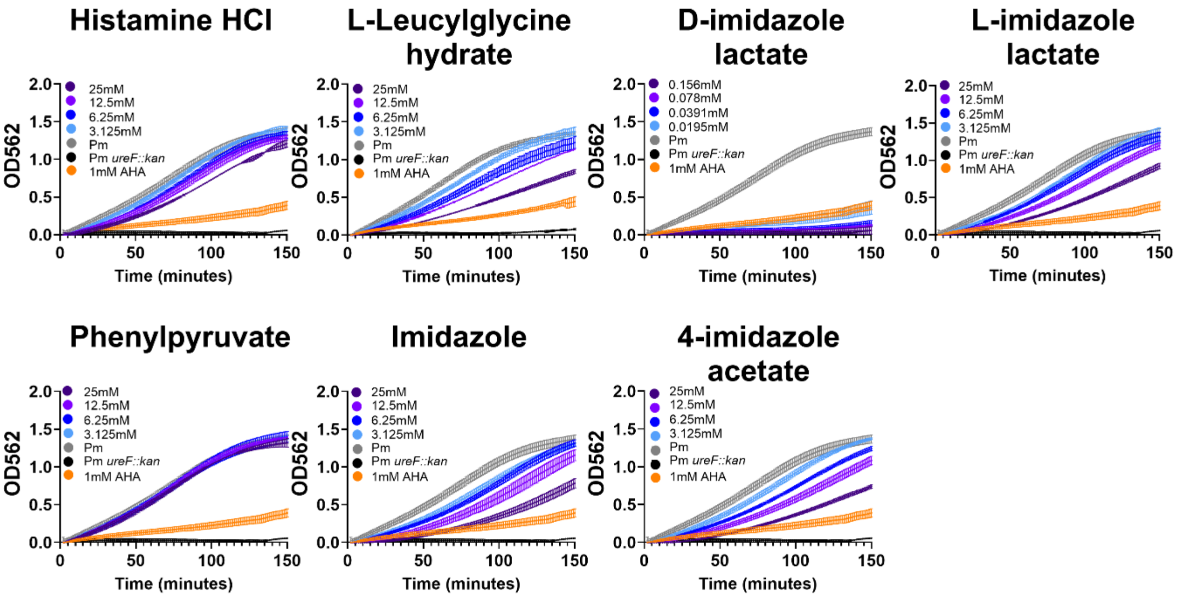

Supplementary Figure 6. All metabolites except phenylpyruvate decrease urease activity in *P. mirabilis* cell free extracts. Representative dose-response curve of urease activity of *P. mirabilis* cell free extracts when incubated in a candidate dampening metabolites or AHA, with a urease mutant (Pm *ureF::kan*) as a negative control. Representative graphs show means and standard deviations from three technical replicates.

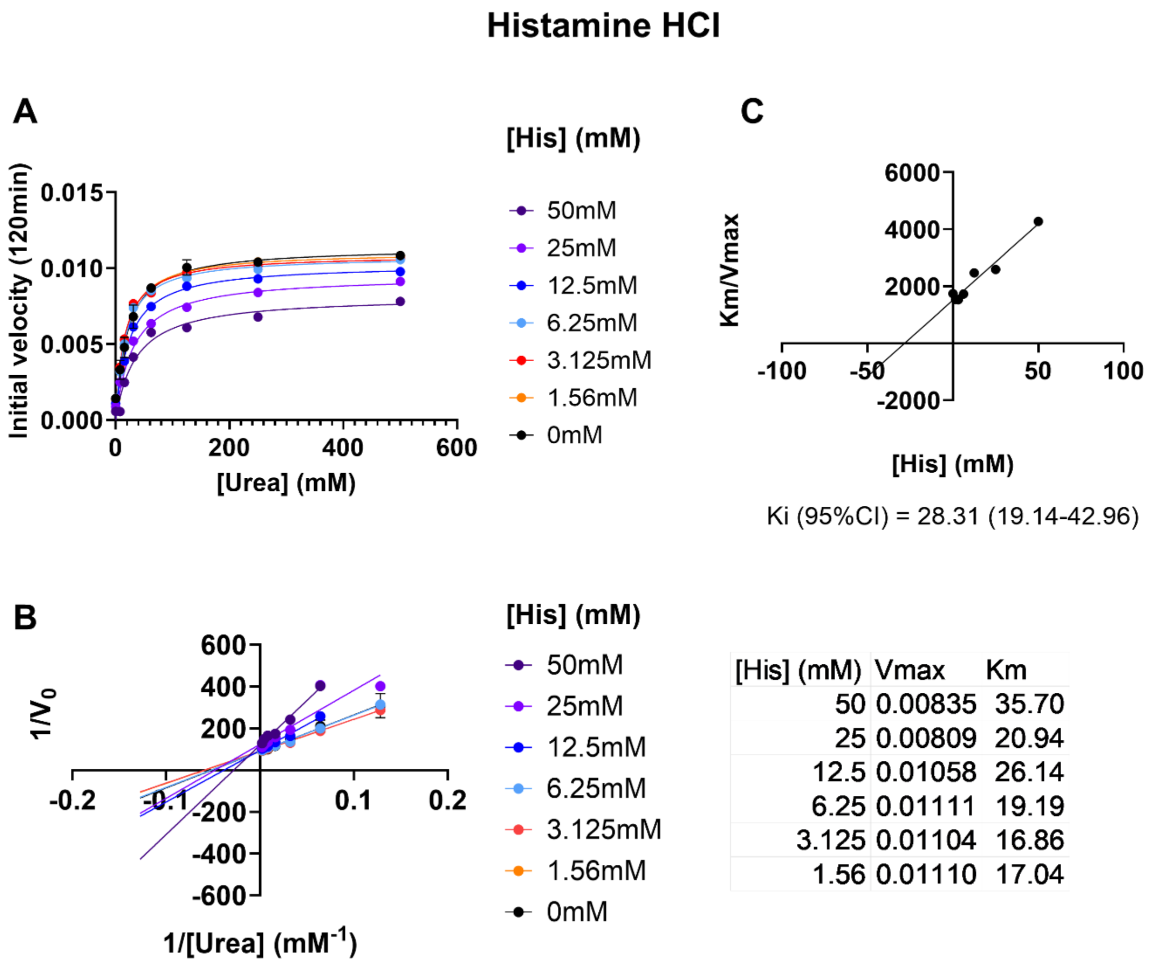

[His] (mM)	Vmax	Km
50	0.00835	35.70
25	0.00809	20.94
12.5	0.01058	26.14
6.25	0.01111	19.19
3.125	0.01104	16.86
1.56	0.01110	17.04

Supplementary Figure 7. **Kinetics of inhibition of urease activity in cell free extracts of *P. mirabilis* by Histamine HCl (His)**. (A) Rates of the reaction as a function of the urea concentration in the reaction mixture in the presence and absence of histamine. (B) Double-reciprocal Lineweaver-Burke plot of the urease activities from panel A (left) and the Vmax and Km values derived from the y-intercepts and x-intercepts respectively of the Lineweaver-Burke plot (right). (C) Plot of the Km/Vmax values derived from panel B as a function of the histamine concentrations. The X-intercept corresponds to -Ki.

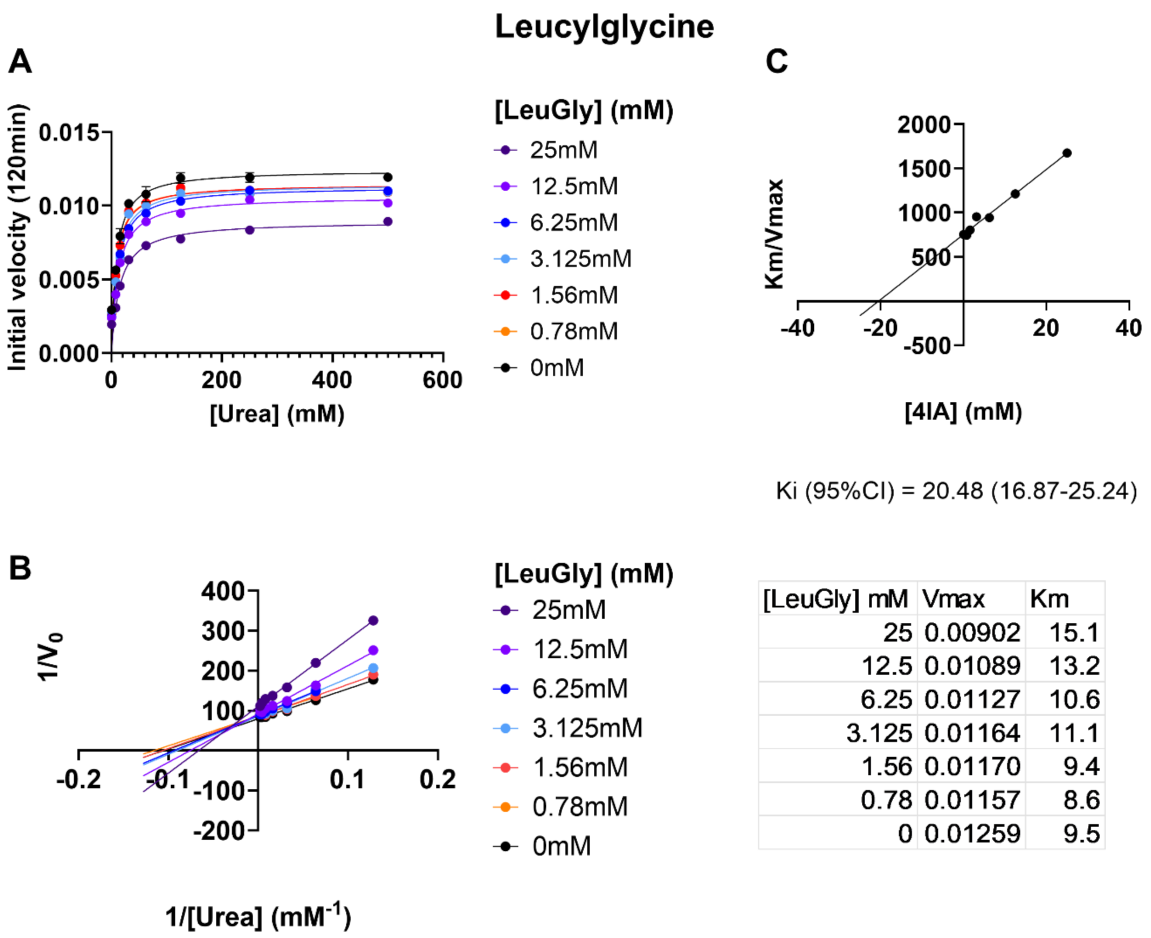

[LeuGly] mM	Vmax	Km
25	0.00902	15.1
12.5	0.01089	13.2
6.25	0.01127	10.6
3.125	0.01164	11.1
1.56	0.01170	9.4
0.78	0.01157	8.6
0	0.01259	9.5

Supplementary Figure 8. **Kinetics of inhibition of urease activity in cell free extracts of *P. mirabilis* by leucylglycine (LeuGly)**. (A) Rates of the reaction as a function of the urea concentration in the reaction mixture in the presence and absence of leucylglycine. (B) Double-reciprocal Lineweaver-Burke plot of the urease activities from panel A (left) and the Vmax and Km values derived from the y-intercepts and x-intercepts respectively of the Lineweaver-Burke plot (right). (C) Plot of the Km/Vmax values derived from panel B as a function of the leucylglycine concentrations. The X-intercept corresponds to -Ki.

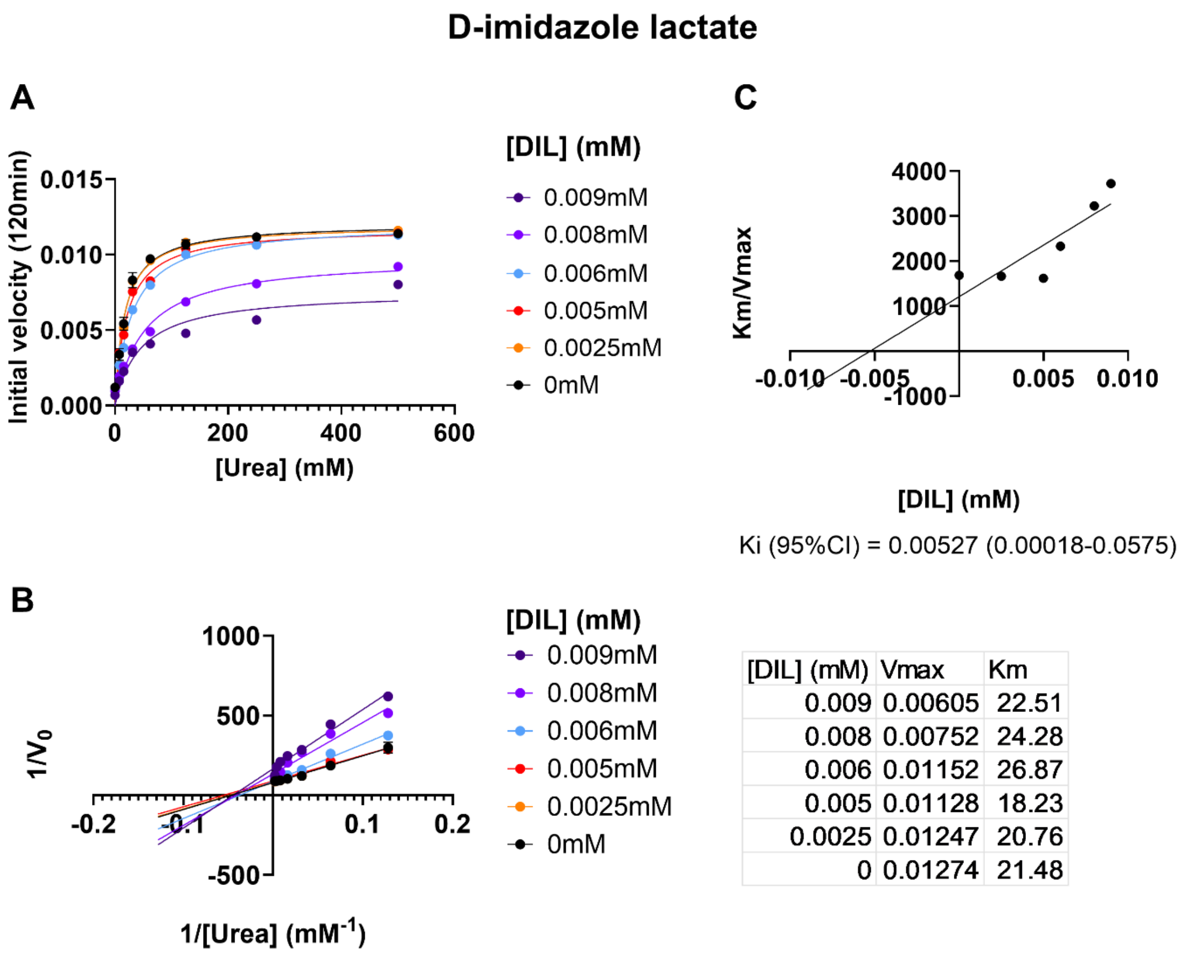

[DIL] (mM)	Vmax	Km
0.009	0.00605	22.51
0.008	0.00752	24.28
0.006	0.01152	26.87
0.005	0.01128	18.23
0.0025	0.01247	20.76
0	0.01274	21.48

Supplementary Figure 9. Kinetics of inhibition of urease activity in cell free extracts of *P. mirabilis* by D-imidazole lactate (DIL). (A) Rates of the reaction as a function of the urea concentration in the reaction mixture in the presence and absence of D-imidazole lactate. (B) Double-reciprocal Lineweaver-Burke plot of the urease activities from panel A (left) and the Vmax and Km values derived from the y-intercepts and x-intercepts respectively of the Lineweaver-Burke plot (right). (C) Plot of the Km/Vmax values derived from panel B as a function of the leucylglycine concentrations. The X-intercept corresponds to -Ki.

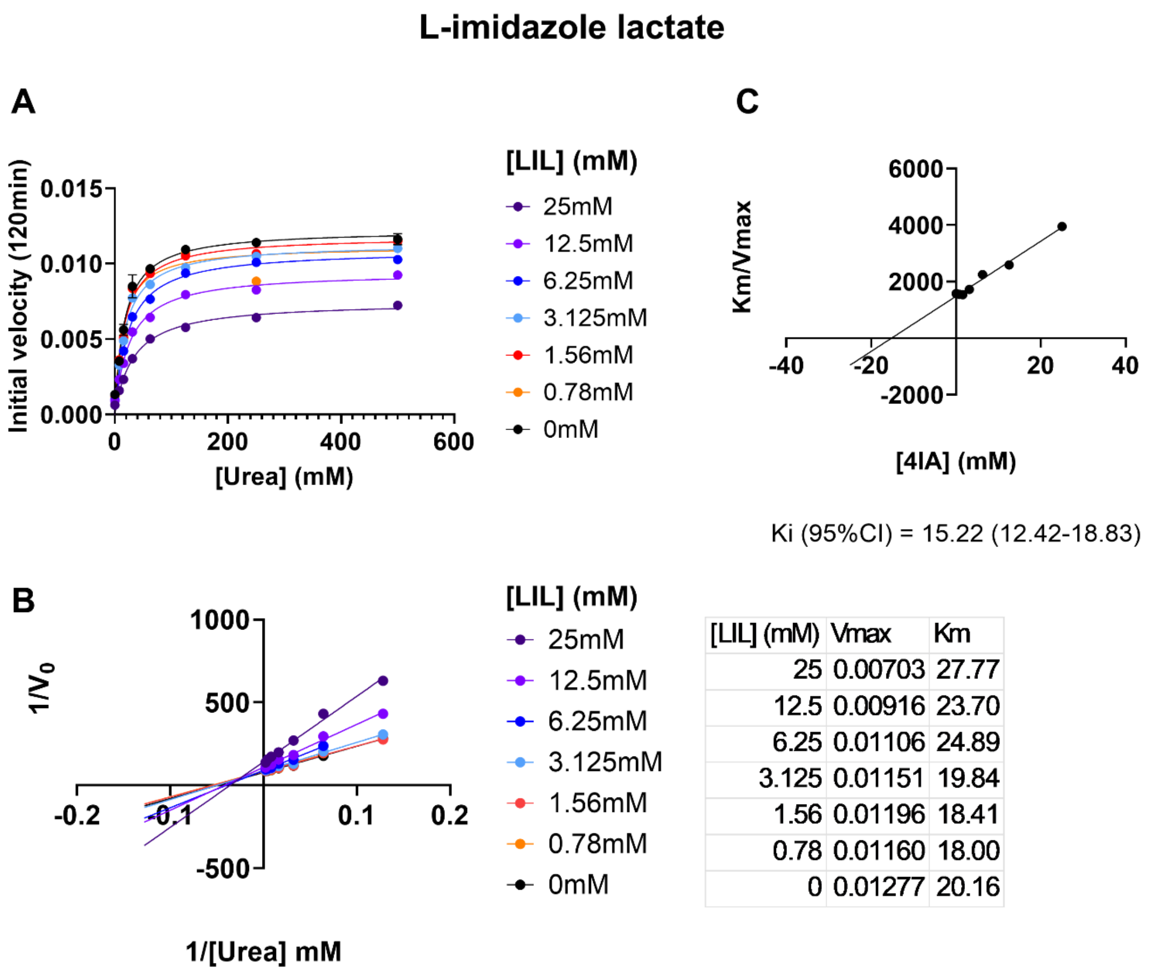

[LIL] (mM)	Vmax	Km
25	0.00703	27.77
12.5	0.00916	23.70
6.25	0.01106	24.89
3.125	0.01151	19.84
1.56	0.01196	18.41
0.78	0.01160	18.00
0	0.01277	20.16

Supplementary Figure 10. Kinetics of inhibition of urease activity in cell free extracts of *P. mirabilis* by L-imidazole lactate (LIL). (A) Rates of the reaction as a function of the urea concentration in the reaction mixture in the presence and absence of L-imidazole lactate. (B) Double-reciprocal Lineweaver-Burke plot of the urease activities from panel A (left) and the Vmax and Km values derived from the y-intercepts and x-intercepts respectively of the Lineweaver-Burke plot (right). (C) Plot of the Km/Vmax values derived from panel B as a function of the leucylglycine concentrations. The X-intercept corresponds to -Ki.

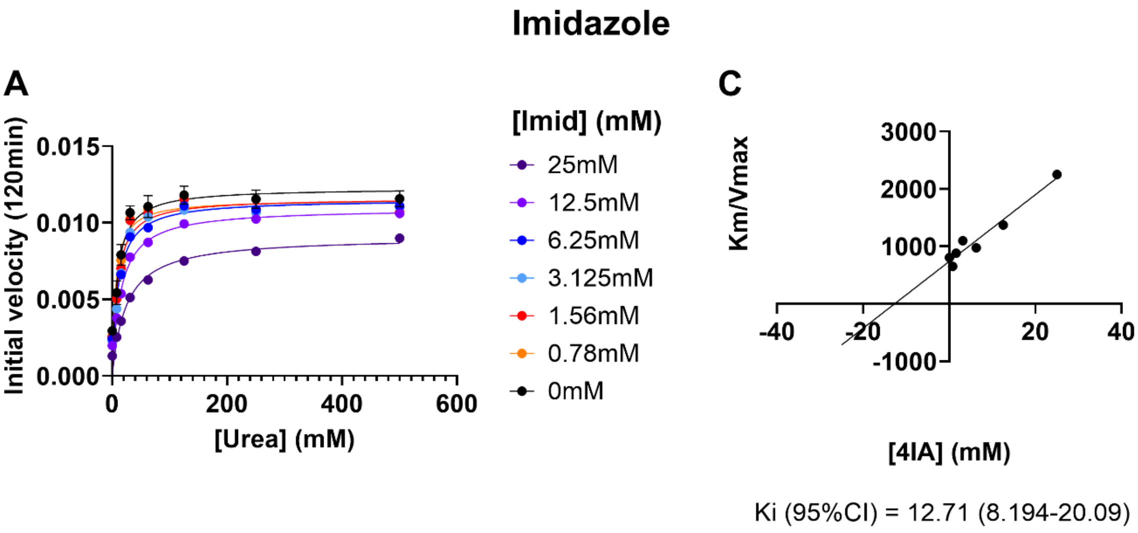

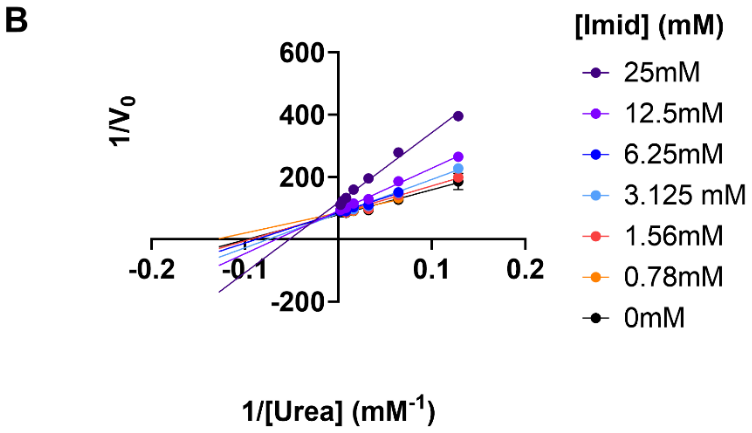

[Imid] mM	Vmax	Km
25	0.00847	19.1
12.5	0.01096	15.0
6.25	0.01168	11.4
3.125	0.01211	13.3
1.56	0.01199	10.6
0.78	0.01172	7.6
0	0.01270	10.2

Supplementary Figure 11. Kinetics of inhibition of urease activity in cell free extracts of *P. mirabilis* by imidazole (Imid). (A) Rates of the reaction as a function of the urea concentration in the reaction mixture in the presence and absence of imidazole. (B) Double-reciprocal Lineweaver-Burke plot of the urease activities from panel A (left) and the Vmax and Km values derived from the y-intercepts and x-intercepts respectively of the Lineweaver-Burke plot (right). (C) Plot of the Km/Vmax values derived from panel B as a function of the leucylglycine concentrations. The X-intercept corresponds to -Ki.

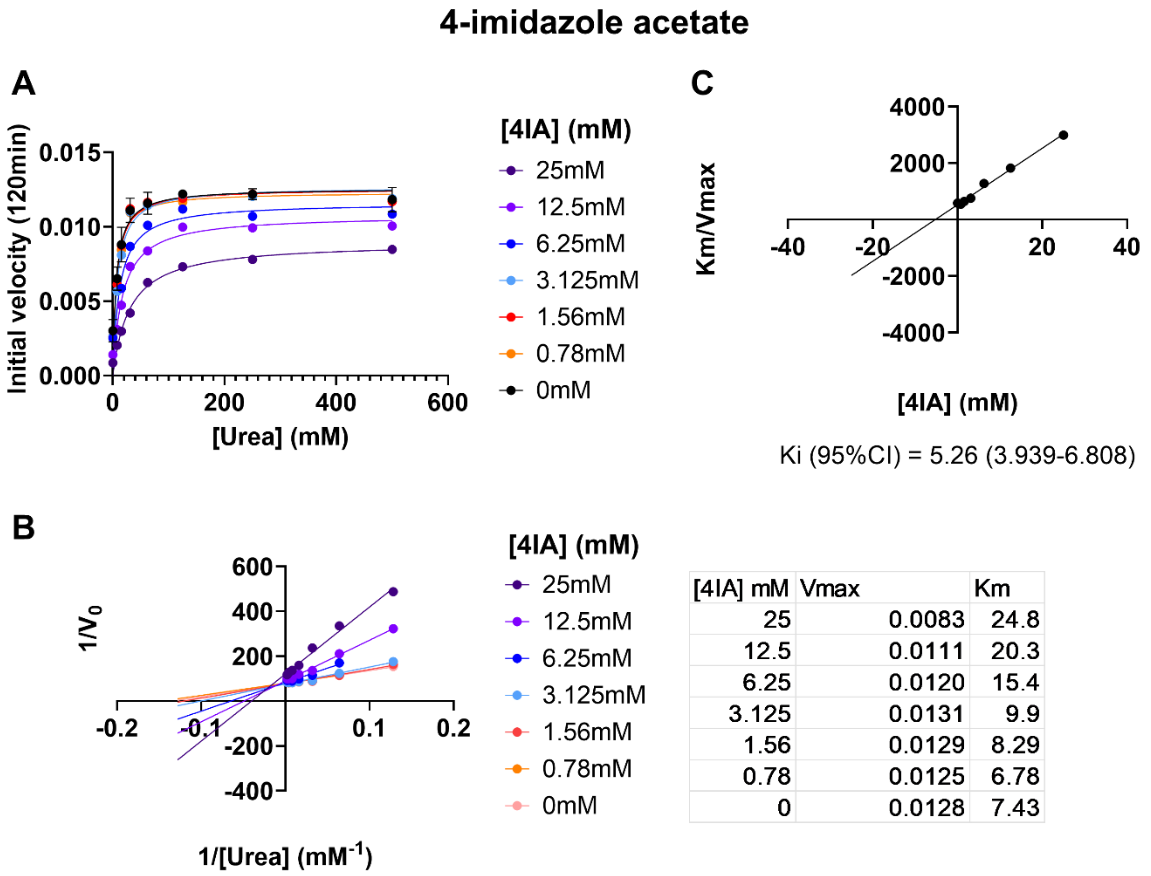

[4IA] mM	Vmax	Km
25	0.0083	24.8
12.5	0.0111	20.3
6.25	0.0120	15.4
3.125	0.0131	9.9
1.56	0.0129	8.29
0.78	0.0125	6.78
0	0.0128	7.43

Supplementary Figure 12. Kinetics of inhibition of urease activity in cell free extracts of *P. mirabilis* by 4-imidazole acetate (4IA). (A) Rates of the reaction as a function of the urea concentration in the reaction mixture in the presence and absence of 4-imidazole acetate. (B) Double-reciprocal Lineweaver-Burke plot of the urease activities from panel A (left) and the Vmax and Km values derived from the y-intercepts and x-intercepts respectively of the Lineweaver-Burke plot (right). (C) Plot of the Km/Vmax values derived from panel B as a function of the leucylglycine concentrations. The X-intercept corresponds to -Ki.

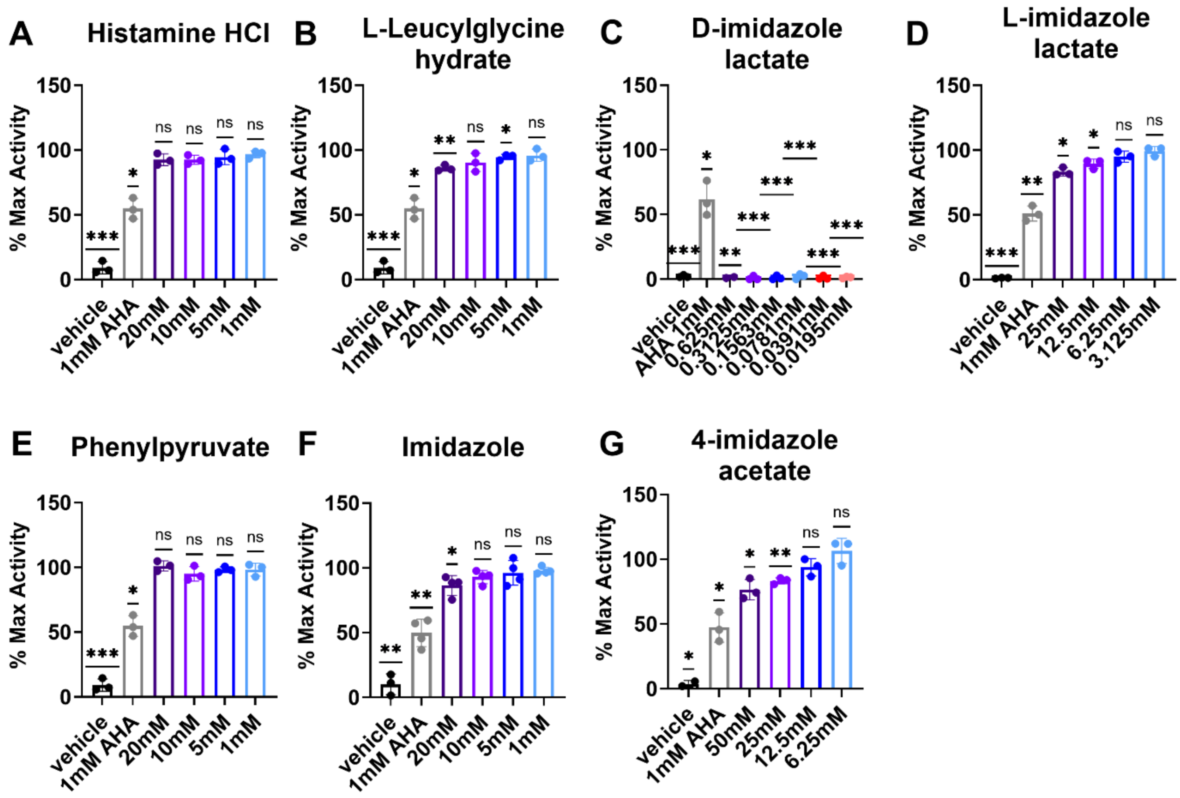

Supplementary Figure 13. Microbial metabolites dampen activity of purified Jack Bean Urease (JBU). 0.1 U of JBU (1U liberates 1 μmol of ammonia from urea per minute) was incubated at 37ºC with shaking in urease-dampening metabolites, 500mM urea, and phenol red. A urea analogue, AHA, served as the positive control for inhibition of JBU. Absorbance (OD562) was measured every 60 seconds for 90 minutes, and activity is expressed as percent of maximum activity (% Max Activity) relative to untreated JBU. Graphs show mean±SD for 3 independent experiments with 3 technical replicates each. Data were analyzed by two tailed one sample t-test to a hypothetical value of 100% activity. ***P<0.001, **P<0.002, *P<0.033.

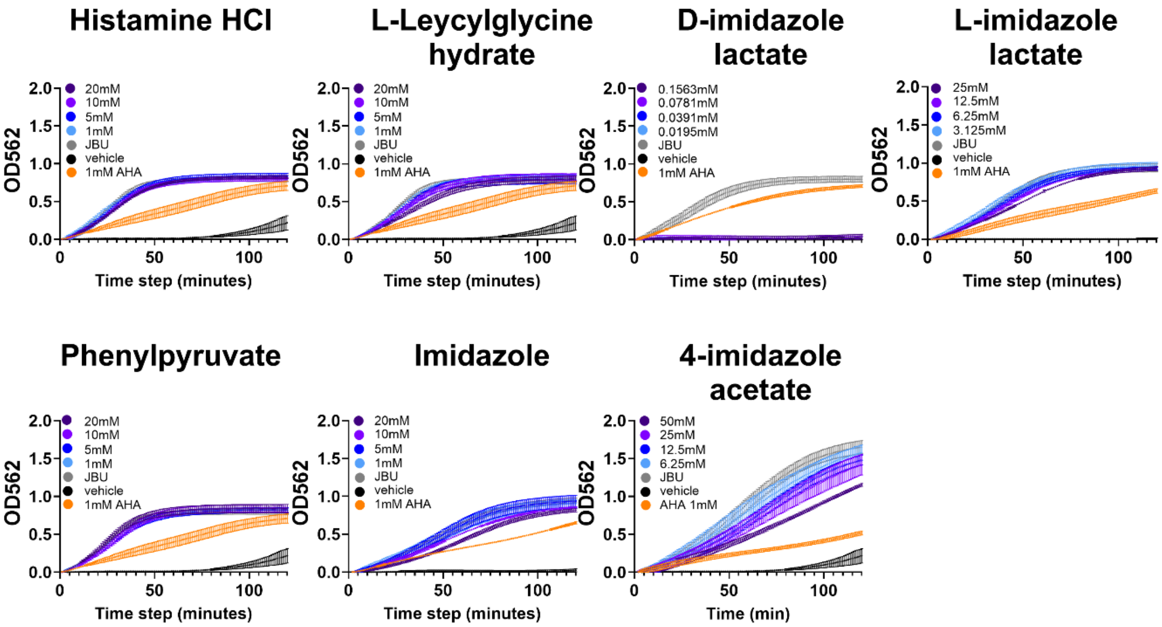

Supplementary Figure 14. JBU activity during incubation in candidate dampening metabolites. Representative dose-response curve of JBU activity when incubated in a candidate dampening metabolites or 1mM AHA, with the vehicle (potassium phosphate buffer pH7) as a negative control. Representative graphs show means and standard deviations from three technical replicates.

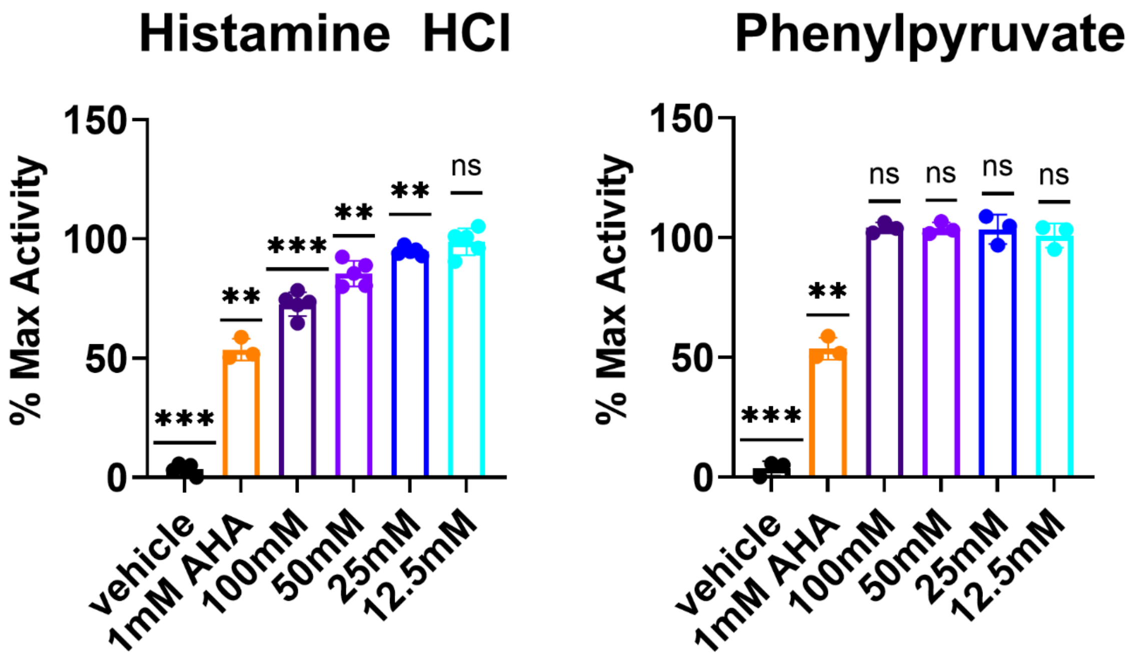

Supplementary Figure 15. Activity of Jack bean urease (JBU) incubated in high concentrations (>20mM) of Histamine HCl and Phenylpyruvate. 0.1 U of JBU (1U liberates 1 μmol of ammonia from urea per minute) was incubated in dampening metabolites supplemented with 500mM urea and phenol red. A urea analogue, AHA, was the positive control. JBU suspensions were incubated at 37ºC with double-orbital shaking in a Synergy H1 plate reader (BioTek), and absorbance (OD562) was measured every 60 seconds for 90 minutes. AUC relative to untreated JBU was compared by two tailed One sample t test to a hypothetical value of 100. ***P<0.001, **P<0.002, *P<0.033. Graphs shows the mean and standard deviation for at least 3 replicates. Each experiment was conducted at least 3 times.

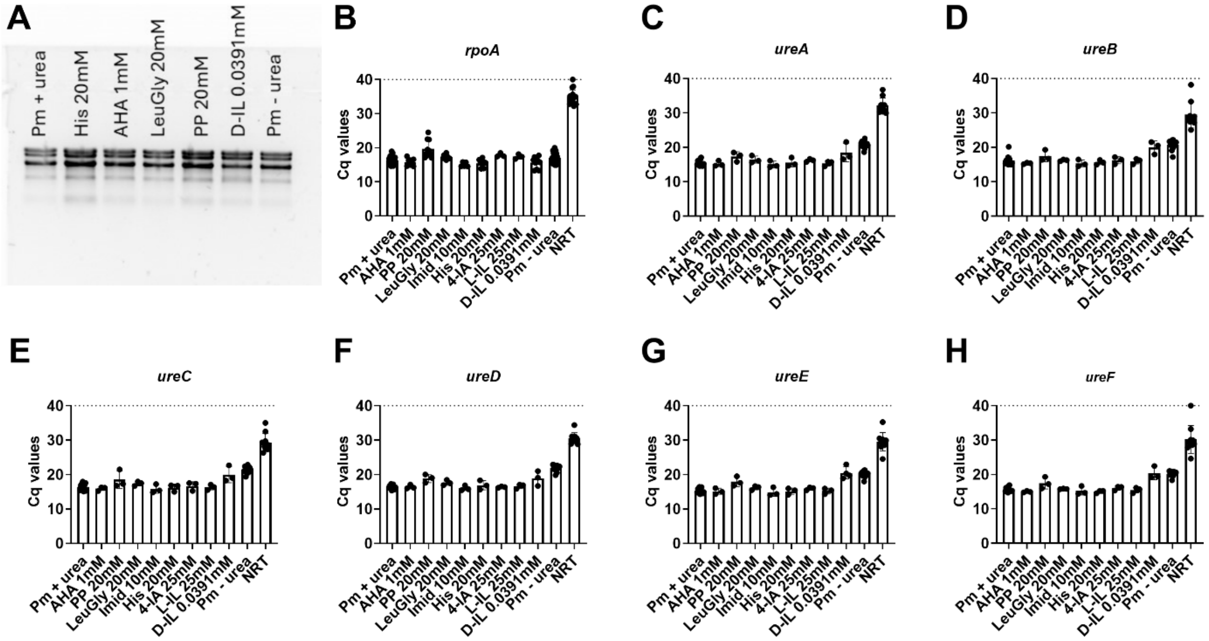

Supplementary Figure 16. RNA transcript stability and expression profiles of *P. mirabilis* urease operon and housekeeping gene. (A) A representative ethidium bromide-stained gel containing RNA isolated from *P. mirabilis* after a 15-minute incubation in buffer alone, buffer with 500mM urea, or buffer with urea and indicated dampening metabolites. Ethidium bromide-stained gels were used to perform RNA integrity analysis. mRNA levels of urease structural genes (*ureABC*), accessory subunit genes involved in nickel incorporation (*ureEDF*), and a housekeeping gene (*rpoA*) were assessed by qRT PCR. Cycle thresholds achieved for (B) the housekeeping gene (*rpoA*) and (C-H) urease operon subunit genes compared to a no-reverse transcriptase control (NRT). Error bars represent mean ± standard deviation (SD) for at least three independent experiments with two technical replicates each.

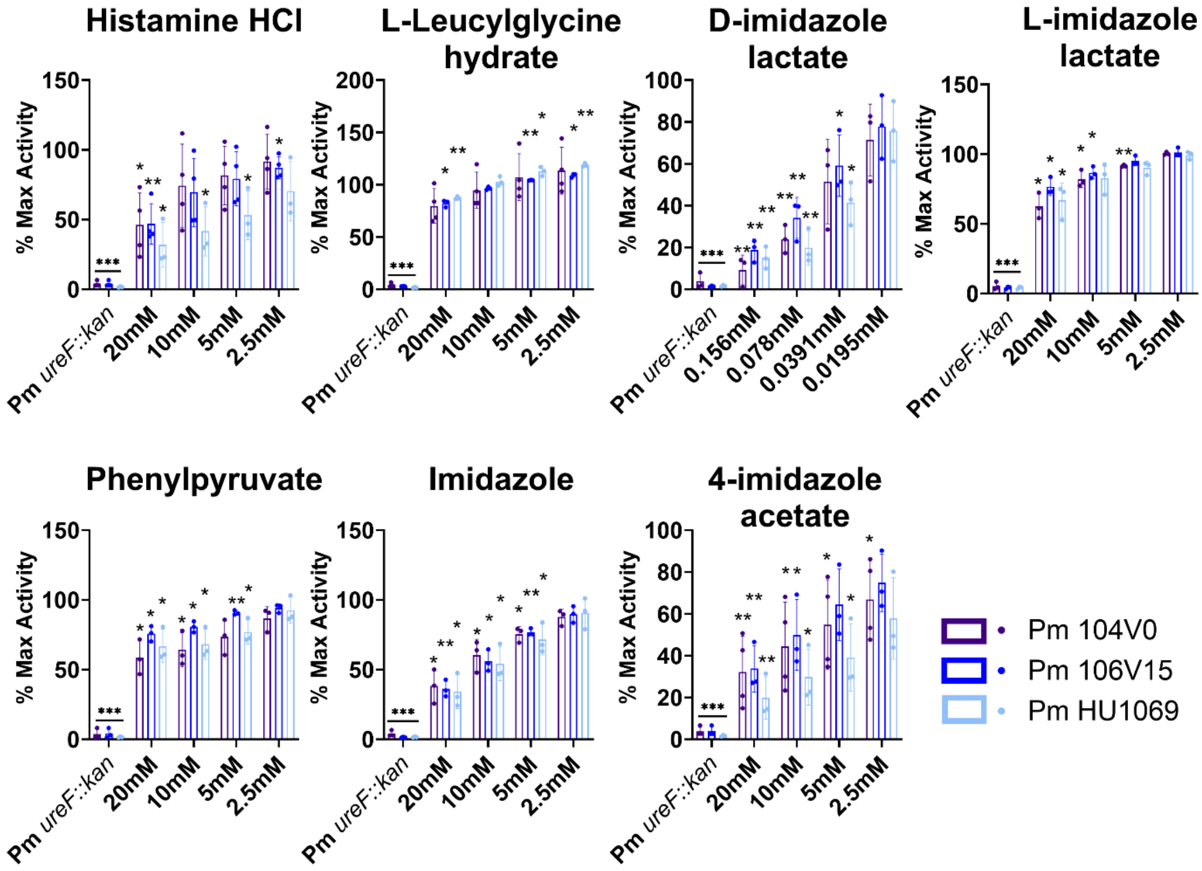

Supplementary Figure 17. Urease activity of *P. mirabilis* 104V0, 106V15 and HU1069 incubated in urease-dampening metabolites. An isogenic *P. mirabilis* HI4320 urease mutant (Pm *ureF::kan*) served as a no-urease control to account for non-urease dependent pH change due to natural breakdown of urea over time. Urease activity is expressed as percent of maximum activity (% Max Activity) relative to the respective untreated *P. mirabilis*. Error bars represent means ± the SD from three independent experiments, with at least three replicates each. ns, not significant (P>0.05); * P<0.033; ** P<0.002; *** P<0.001 (as determined by two tailed One sample t test to a hypothetical value of 100).

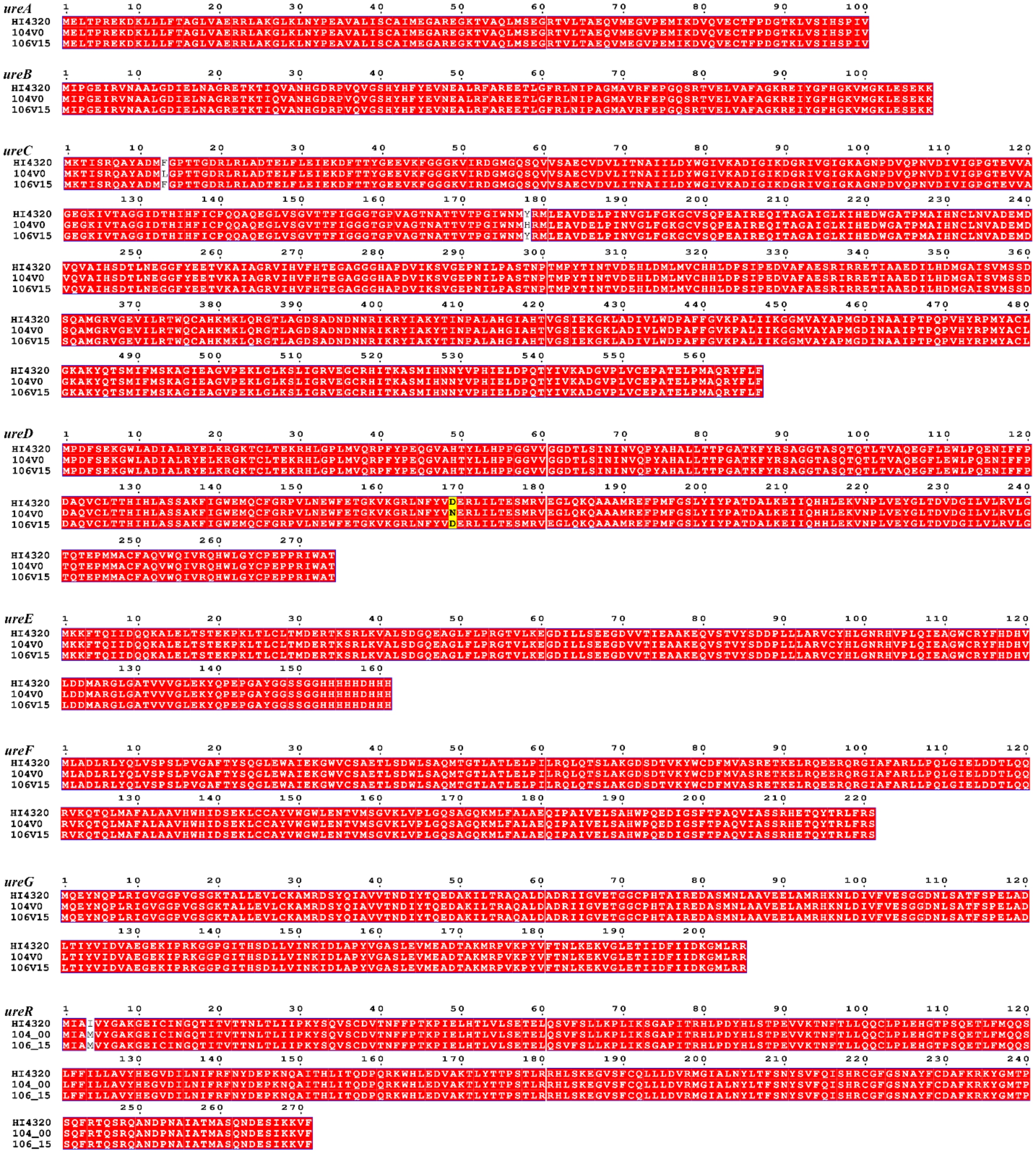

Supplementary Figure 18. Multiple sequence alignment of urease protein subunits across three *Proteus mirabilis* isolates. Amino acid sequences of urease subunits from strains HI4320, 104V0, and 106V15 were aligned using Clustal Omega and visualized using ESPript 3.0 (*69*). The colors in the alignment output indicate sequence conservation and identity. Red: highly conserved

residues (strictly identical across sequences), yellow: similar residues (partially conserved based on biochemical properties), white (no color): non-conserved residues or gaps in the alignment.

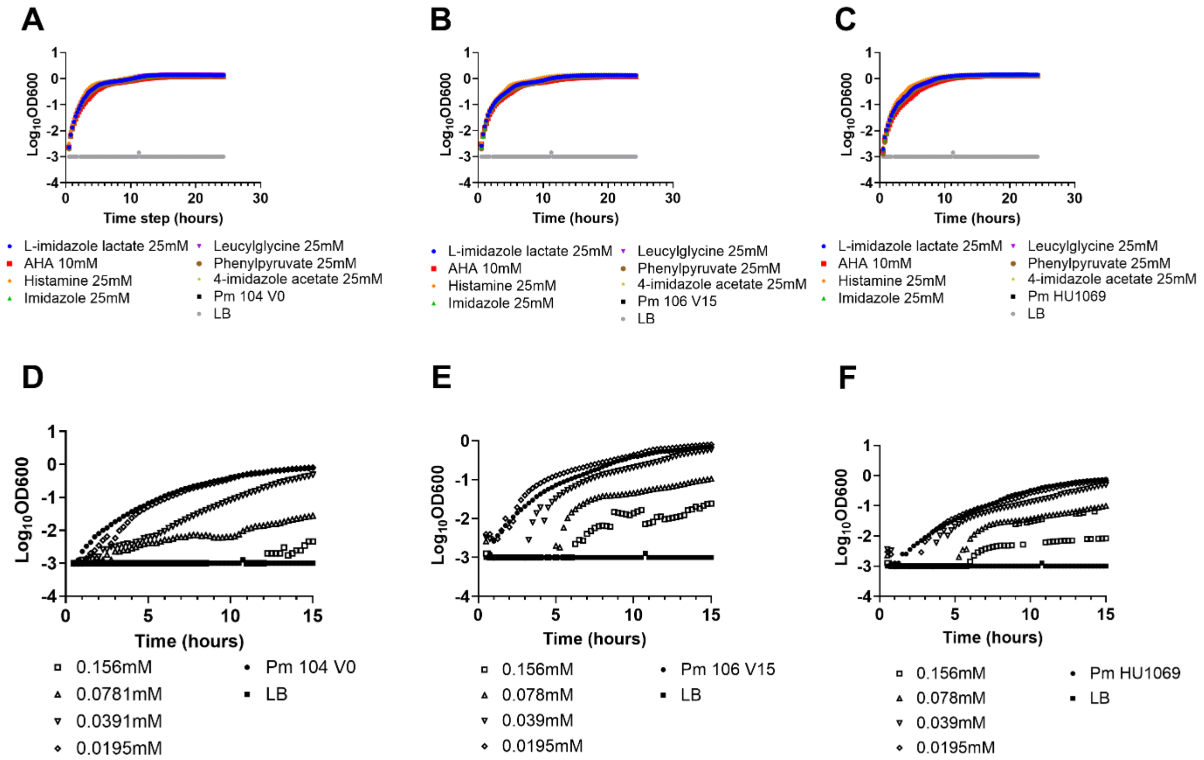

Supplementary Figure 19. Growth of clinical isolates of *P. mirabilis* incubated in dampening metabolites. *P. mirabilis* clinical isolates 104V0 (A,D), 106V15 (B,E), and HU1060 (C,F) were incubated in candidate dampening metabolites to assess growth. *P. mirabilis* was cultured for ~18 hours in LB broth, then diluted 1:100 into fresh LB broth with (A-C) the highest concentrations of dampening metabolites tested for urease inhibition or (D-F) serial dilutions of D-imidazole lactate. Bacterial suspensions were incubated at 37ºC with double-orbital shaking, and OD600 was measured every 15 minutes for 18 hours. Error bars represent mean ± standard deviation (SD) from at least 3 technical replicates, and graphs are representative of 3 independent biologic experiments.

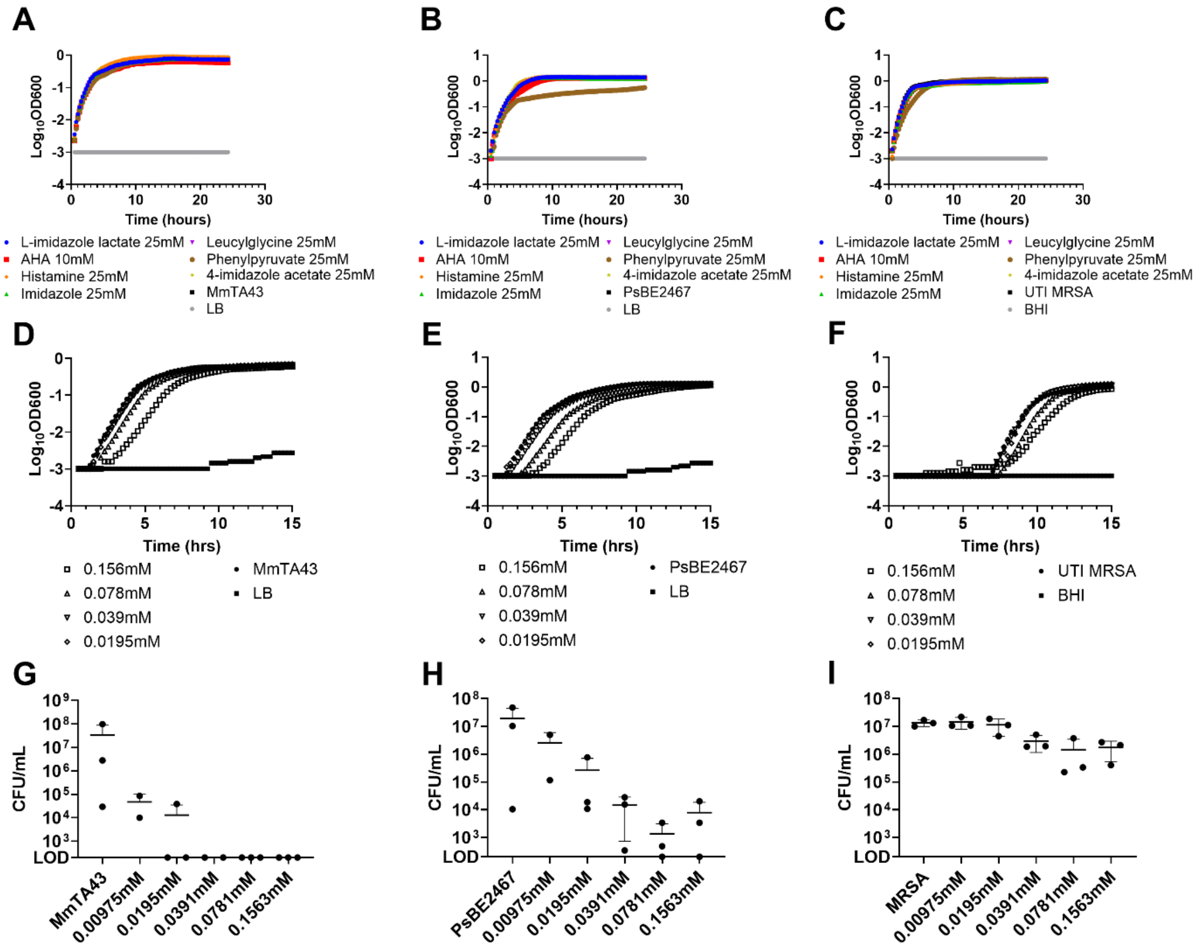

Supplementary Figure 20. Growth of *M. morganii, P. stuartii* and UTI MRSA incubated in dampening metabolites. *M. morganii* TA43 (A,D,G), *P. stuartii* BE2467(B,E,H), UTI MRSA (C,F,I) were incubated in urease dampening metabolites to assess impact on growth. Bacteria were cultured for ~18 hours in LB (*Mm* and *Ps*) or BHI (MRSA) broth, then diluted 1:100 into fresh broth with (A-C) the highest concentrations of dampening metabolites assessed for urease inhibition or (D-F) serial dilutions of D-imidazole lactate. Bacterial suspensions were incubated at 37ºC with double-orbital shaking, and OD600 was measured every 15 minutes for 18 hours. Error bars represent mean ± standard deviation (SD) from at least 3 technical replicates, and graphs are representative of 3 independent biologic experiments. (G-I) Viability of incubated in *Mm, Ps* or MRSA incubated in serial dilutions of D-imidazole lactate. Overnight cultures were

subcultured to mid log phase, washed in potassium phosphate buffer, and diluted 1:10 in potassium phosphate buffer with and without D-imidazole lactate. Samples (n=3) were incubated at 37°C with aeration for one hour then plated and enumerated for colony forming units (CFU). Error bars represent mean ± standard deviation (SD) from three biologic replicates.

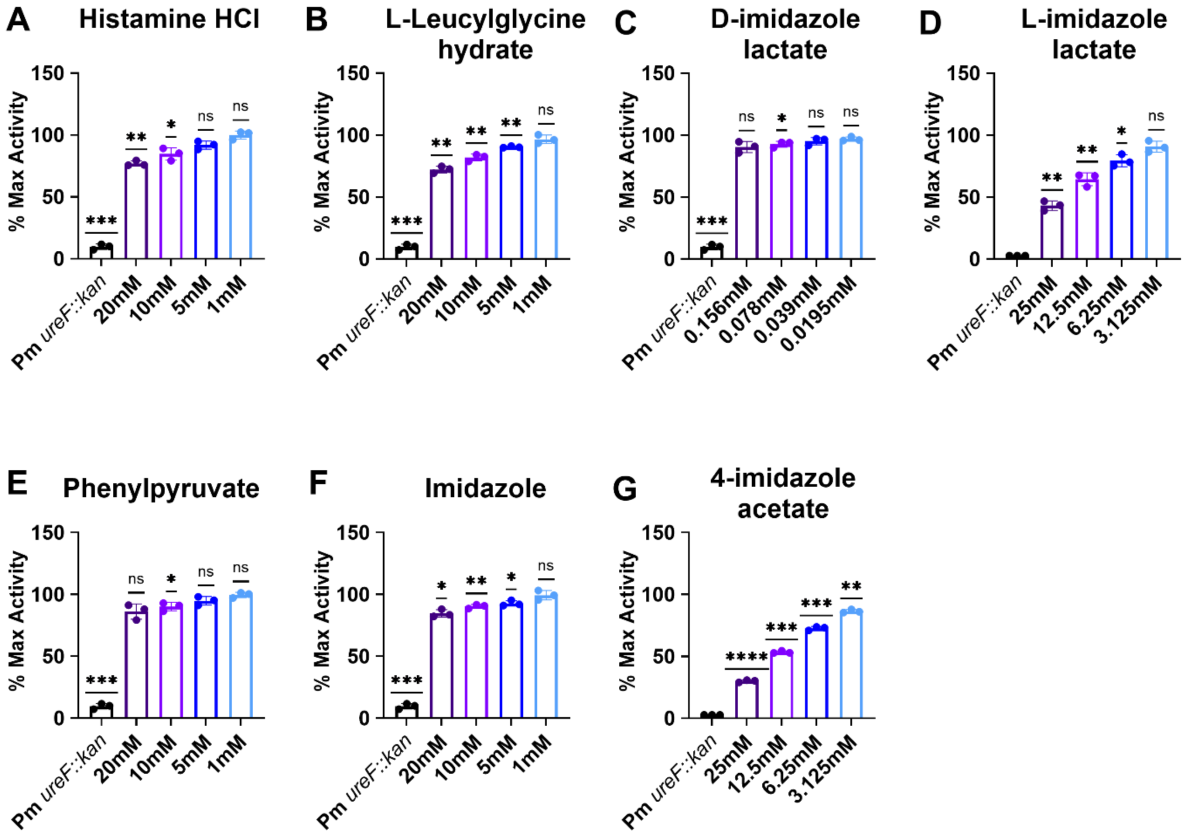

Supplementary Figure 21. Microbial metabolites dampen *P. mirabilis* urease activity in human urine. Urease activity of Pm incubated in candidate urease-dampening metabolites in human urine**.** Activity from each 180-minute assay was expressed as Area Under the Curve (AUC) and adjusted to the percent of maximum activity (% Max Activity) relative to untreated Pm and analyzed by one sample t-test to a hypothetical value of 100%. All error bars represent mean±SD from three independent experiments, with three replicates each. ns, not significant ($P>0.05$); * $P<0.033$; ** $P<0.002$); *** $P<0.001$

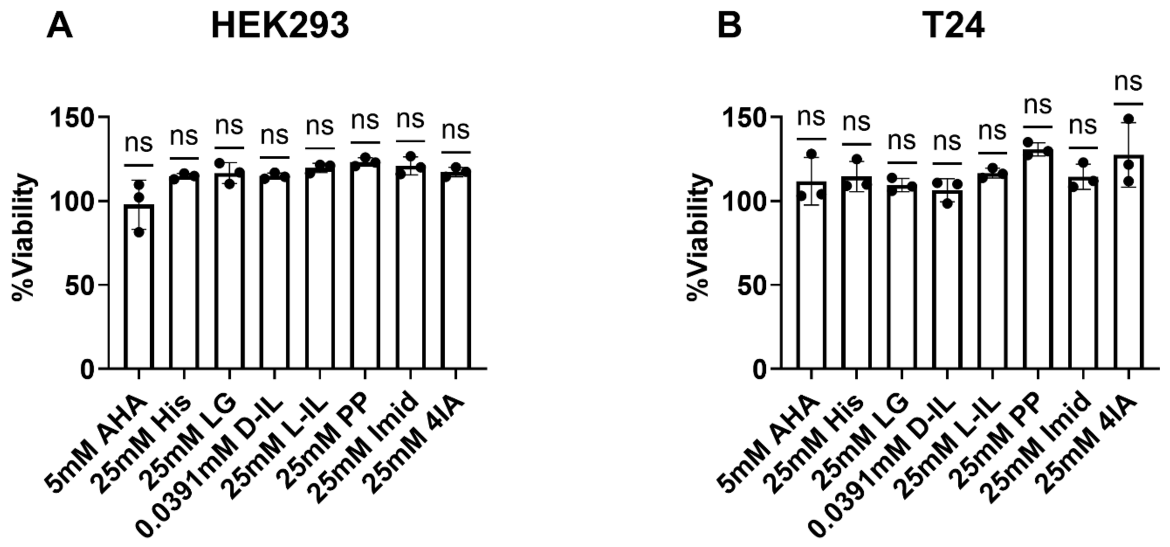

Supplementary Figure 22. Urease-dampening metabolites are non-cytotoxic. (A-B) Lactate dehydrogenase cytotoxicity assay measuring the viability of (A) human embryonic kidney cells (HEK293) cells and (B) human bladder epithelial cells (T24) incubated in urease dampening metabolites for 24 hours. Triton X-100 was used as positive control for cell lysis. All error bars represent mean±SD from three independent experiments, with two replicates each. ns, not significant (P>0.05); * P<0.033; ** P<0.002); *** P<0.001

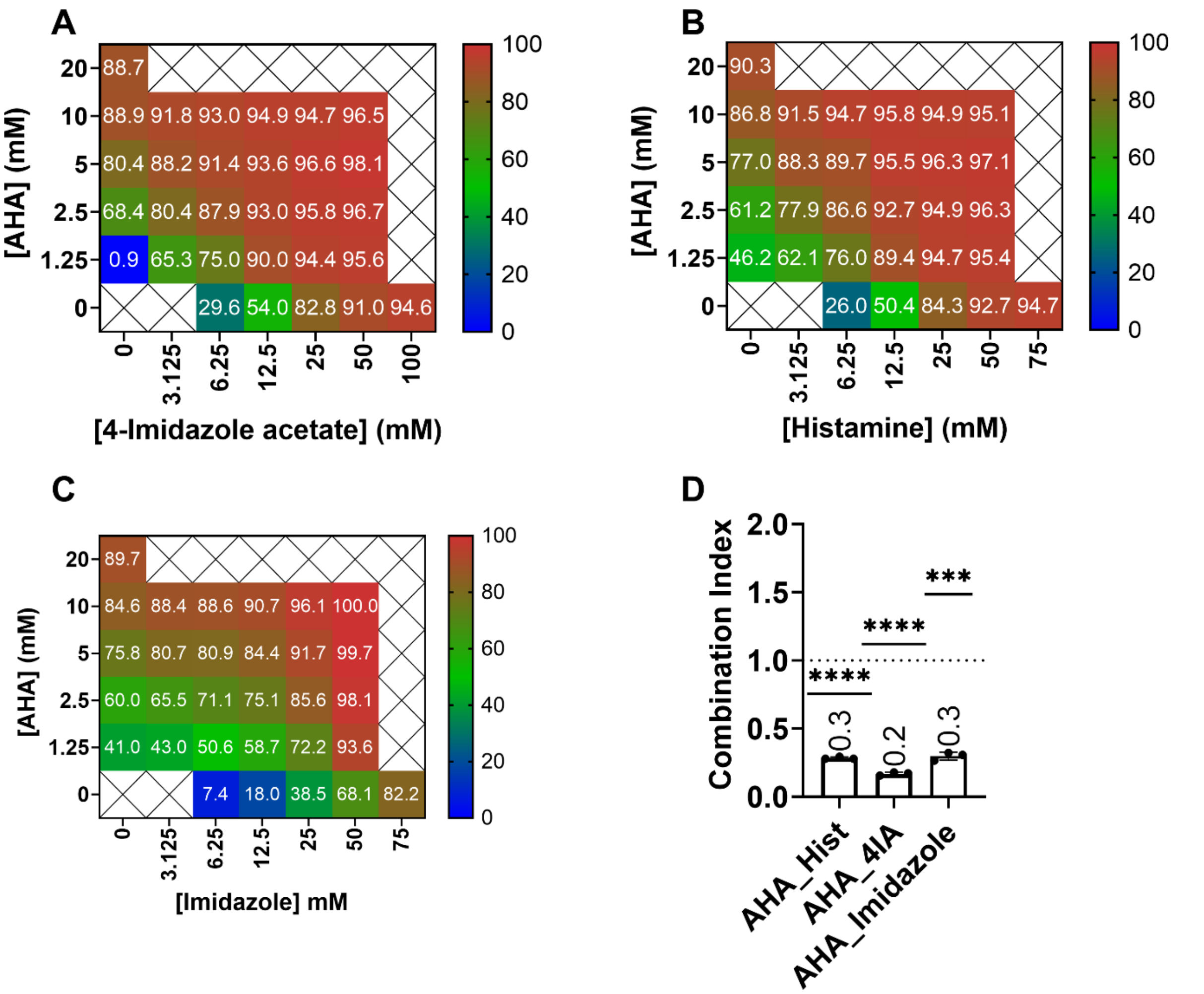

Supplementary Figure 23. Synergistic effects of Histamine or 4-imidazole acetate, in combination with AHA and alone, on *P. mirabilis* urease activity in artificial urine media (AUM) supplemented with 500mM urea. Urease activity is expressed as the mean percent of inhibition (calculated by AUC) relative to untreated *P. mirabilis*. (A) Representative heat map of the mean percent inhibition of *P. mirabilis* urease activity when incubated in AHA and Histamine in combination and alone in AUM with 500mM urea. (B) Representative heat map of the mean percent inhibition of *P. mirabilis* urease activity when incubated in AHA and 4-imidazole acetate in combination and alone in AUM with 500mM urea. (C) Representative heat map of the mean percent inhibition of *P. mirabilis* urease activity when incubated in AHA and

imidazole in combination and alone in AUM with 500mM urea. (D) Combination index (CI) of AHA in combination with histamine, 4-imidazole acetate and imidazole respectively. CI was calculated using the concentrations of urease-dampening metabolites A and B that achieve the desired effect (40-80% inhibition of *P. mirabilis* urease activity) in combination and alone. One sample T test compared CI to the hypothetical of CI=1 (additive effect). ***$P<0.001$, **$P<0.002$, *$P<0.01$.

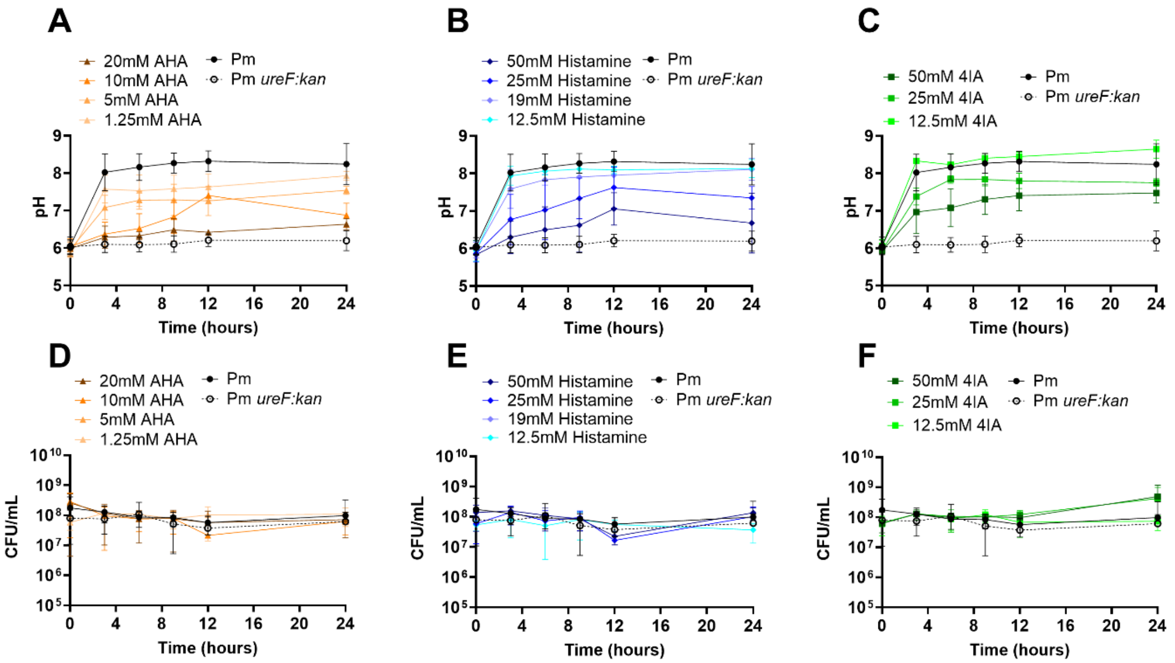

Supplementary Figure 24. Dose response of pH and bacterial CFUs to AHA, histamine, and 4-imidazole acetate in the *in vitro* CAUTI model. An initial bacterial inoculum of 10^8 CFU of *P. mirabilis* or *P. mirabilis ureF::kan* was added to AUM in the "bladder" inner chamber, followed by initiation of flow and regular effluent collection over the 24-hour study period. (A-C) pH and (D-F) CFUs of *P. mirabilis* were enumerated from the effluent collected through the catheter port for each "bladder" condition at 0, 3, 6, 9, 12, and 24 hours post inoculation. Data represent mean ± SD for at least three independent glass "bladder" experiments (Pm n=18, Pm *ureF::kan* n=18, all other conditions n=3). Acetohydroxamic acid (AHA), histamine (Hist), 4-imidazole acetate (4-IA).

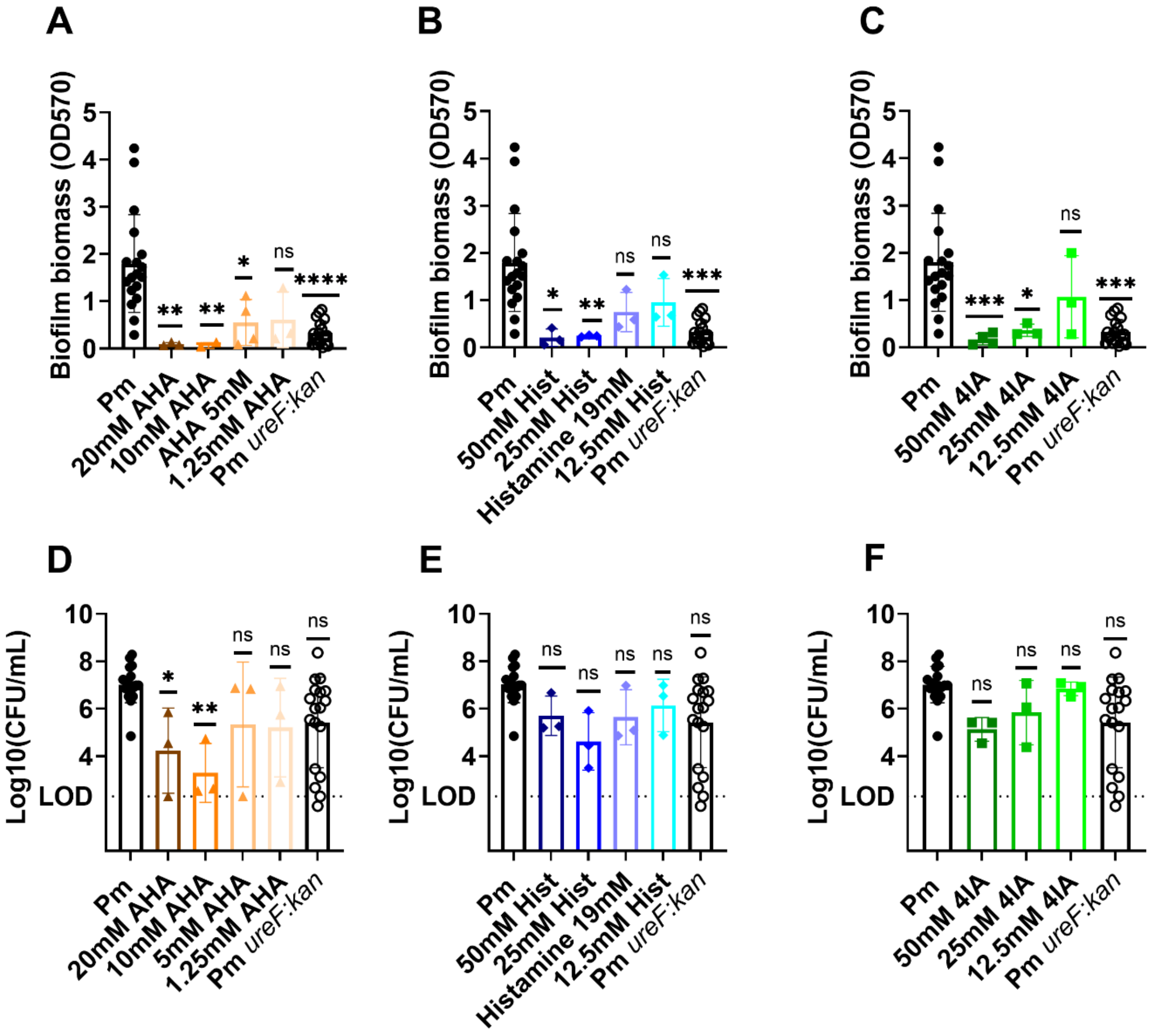

Supplementary Figure 25. Measuring bacterial crystalline biofilm biomass and bacterial viability on 10 mm catheter segments 24 hours post-inoculation. An initial bacterial inoculum of 10^8 CFU of *P. mirabilis* or *P. mirabilis ureF::kan* was added to AUM in the "bladder" inner chamber, followed by initiation of flow and regular effluent collection over the 24-hour study period. Catheter biofilms were assessed by crystal violet staining **(A-C)** and CFU quantification **(D-F)** on 10 mm catheter segments 24 hours post-inoculation. Data represents mean + SD for at least three independent glass "bladder" experiments with three replicate catheter segments each (Pm n=18, Pm *ureF::kan* n=18, all other conditions n=3). Statistical analysis performed was

One-way ANOVA with Dunnett’s multiple comparisons (each condition compared to Pm alone), ns = non-significant, * $p < 0.05$, ** $p < 0.01$, *** $p < 0.001$, **** $p < 0.0001$.

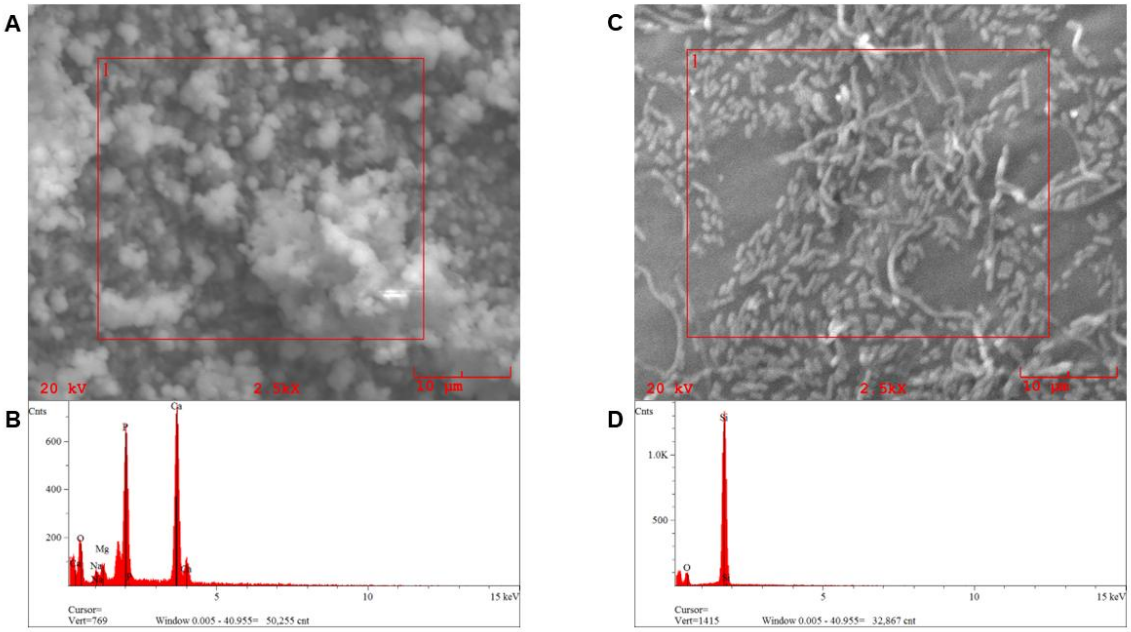

Supplementary Figure 26. Elemental composition of crystalline biofilm measured by energy dispersive spectroscopy (EDS). Representative SEM images of *P. mirabilis* (A) and *P. mirabilis ureF::kan* (C) biofilms formed on the lumen of catheter eyelets collected from an *in vitro* CAUTI model. Regions subjected to EDS analysis are indicated with the red box. EDS spectra derived from crystalline structures observed within *Proteus mirabilis* (B) predominantly show calcium (CA), phosphate (P), magnesium (Mg), oxygen (O), while spectra from *P. mirabilis ureF::kan* (D) do not contain any ion precipitates and only show silicon (Si) from the catheter surface and oxygen (O).

60. . (LibreTexts™ CHEMISTRY).
61. P. G. Wenthold. (LibreTexts™ CHEMISTRY), pp. 22.03.21.
62. A. M. Bolger, M. Lohse, B. Usadel, Trimmomatic: a flexible trimmer for Illumina sequence data. *Bioinformatics* **30**, 2114-2120 (2014).
63. A. Bankevich *et al.*, SPAdes: a new genome assembly algorithm and its applications to single-cell sequencing. *Journal of computational biology* **19**, 455-477 (2012).
64. R. R. Wick, L. M. Judd, C. L. Gorrie, K. E. Holt, Unicycler: resolving bacterial genome assemblies from short and long sequencing reads. *PLoS computational biology* **13**, e1005595 (2017).
65. A. Bourgogne *et al.*, Large scale variation in Enterococcus faecalis illustrated by the genome analysis of strain OG1RF. *Genome biology* **9**, 1-16 (2008).
66. B. Langmead, S. L. Salzberg, Fast gapped-read alignment with Bowtie 2. *Nature methods* **9**, 357-359 (2012).
67. H. Li *et al.*, The sequence alignment/map format and SAMtools. *bioinformatics* **25**, 2078-2079 (2009).
68. T. Seemann, Prokka: rapid prokaryotic genome annotation. *Bioinformatics* **30**, 2068-2069 (2014).
69. X. Robert, P. Gouet, Deciphering key features in protein structures with the new ENDscript server. *Nucleic Acids Research* **42**, W320-W324 (2014).
70. T. Brooks, C. Keevil, A simple artificial urine for the growth of urinary pathogens. *Letters in applied microbiology* **24**, 203-206 (1997).
71. J. A. Carter, A. I. Barros, J. A. Nóbrega, G. L. Donati, Traditional Calibration Methods in Atomic Spectrometry and New Calibration Strategies for Inductively Coupled Plasma Mass Spectrometry. *Frontiers in Chemistry* **6**, (2018).
72. G. L. Long, J. D. Winefordner, Limit of Detection A Closer Look at the IUPAC Definition. *Analytical Chemistry* **55**, 712A-724A (1983).
