# Supplementary material for: Harnessing microbial-derived metabolites in the urinary tract to prevent infection induced catheter encrustation
Source: Nat Commun. 2025 Nov 3;16:9678. doi: 10.1038/s41467-025-64661-y (PMC12583827; doi:10.1038/s41467-025-64661-y)
Supplement: Supplementary file 2 — Description of Additional Supplementary Files [file 41467_2025_64661_MOESM2_ESM.pdf]

Title: Supplementary Data 1
Description: A complete list of bacterial strains used in this study.

Title: Supplementary Data 2
Description: Prioritized metabolites to screen for urease inhibition activity.

Title: Supplementary Data 3
Description: Additional compounds screened that shared functional groups and/or on the same biosynthetic pathways to the verified metabolites.

Title: Supplementary Data 4
Description: Calculated J couplings of Peaks in variable temperature (VT) NMR spectra obtained with the solvent dimethyl sulfoxide-d6 (DMSO-d6) and J couplings of Peaks in NMR spectrum with the solvent deuterium dioxide (D2O). Both temperature-induced rotation and water-induced tautomerization support the assertion that the imidazole ring is restricted in its motion and that one rotamer is expected to dominate for each of the D- and L- isomers of imidazole lactic acid.

Title: Supplementary Data 5

Description: Combination index calculations for each pairwise combination.

Title: Supplementary Data 6
Description: ICP-EOS p values. Data were analyzed by ordinary one-way ANOVA with Dunnett's multiple comparisons.

Title: Supplementary Data 7
Description: Candidate global metabolites contributing to modulation of *Proteus mirabilis* urease activity

Title: Supplementary Data 8
Description: ICP-OES operating conditions used for Ca, Mg and P determination in catheter deposit samples.

Title: Supplementary Data 9
Description: Addition and recovery experiments to evaluate the determination of Ca, Mg and P in acid-digested samples of catheter deposits by ICP-OES. Percent recovery results are shown as the mean ± 1 standard deviation (%, n = 3).
